# Physiological responses to capture, handling and tagging in the critically endangered flapper skate (*Dipturus intermedius*)

**DOI:** 10.1093/conphys/coae077

**Published:** 2024-11-28

**Authors:** Georgina Cole, Edward Lavender, Adam Naylor, Simon Girling, Dmitry Aleynik, Steffen Oppel, Jane Dodd, James Thorburn

**Affiliations:** Conservation Department, Royal Zoological Society of Scotland, 134 Corstorphine Road, Edinburgh, Scotland, EH12 6TS, UK; Centre for Research into Ecological and Environmental Modelling, University of St Andrews, The Observatory, Buchanan Gardens, St. Andrews, Scotland, KY16 9LZ, UK; Scottish Oceans Institute, University of St Andrews, Gatty Marine Laboratory, Institiud Chuantan na h-Alba, East Sands, St Andrews, Scotland, KY16 8LB, UK; Department of Systems Analysis, Integrated Assessment and Modelling, Eawag, Swiss Federal Institute of Aquatic Science and Technology, Überlandstrasse 133, Dübendorf CH-8600, Switzerland; Conservation Department, Royal Zoological Society of Scotland, 134 Corstorphine Road, Edinburgh, Scotland, EH12 6TS, UK; New Zealand Centre for Conservation Medicine, Auckland Zoo, 91 Motions Road, Western Springs, Auckland 1022, New Zealand; Conservation Department, Royal Zoological Society of Scotland, 134 Corstorphine Road, Edinburgh, Scotland, EH12 6TS, UK; Scottish Association for Marine Science, Dunbeg, Oban, Argyll, Scotland, PA37 1QA, UK; Swiss Ornithological Institute, Seerise 1, 6204 Sempach, Switzerland; Nature Scot, Cameron House, Albany Street, Oban, Scotland, PA34 4AE, UK; Scottish Oceans Institute, University of St Andrews, Gatty Marine Laboratory, Institiud Chuantan na h-Alba, East Sands, St Andrews, Scotland, KY16 8LB, UK; School of Applied Sciences, Edinburgh Napier University, 9 Sighthill Court, Edinburgh, Scotland, EH11 4BN, UK; Centre for Conservation and Restoration Science, Edinburgh Napier University, 9 Sighthill Court, Edinburgh, Scotland, EH11 4BN, UK

**Keywords:** Acidosis, angling, batoid, conservation, *Dipturus intermedius*, elasmobranch, flapper skate, physiology, tagging

## Abstract

Catch-and-release angling is a popular recreational pastime and an essential component of many fish research programmes. Marked physiological disturbances have been documented in elasmobranchs in response to angling and handling, but skates and rays remain understudied. Here, we describe for the first time the physiological responses of the critically endangered flapper skate (*Dipturus intermedius*) to angling, handling and tagging in Scotland. Sixty-one skate were captured by angling as part of a tagging research programme. We assessed individual health, measured blood parameters at two time points (post-capture and prior to release) and recorded heart and respiratory rates during handling and the surgical insertion of acoustic tags. Injuries or infections were identified in 10% of individuals and attributed to prior angling in two cases. Skate generally experienced a mild metabolic acidosis characterized by decreases in blood pH and bicarbonate and increases in lactate and glucose. Respiratory acidosis characterized by limited increases in PCO_2_ was also observed. The degree of acidosis was greater with warmer sea temperatures and longer fight times, and worsened during the time that skate were handled on deck. Heart rates during handling were negatively associated with body size, positively associated with temperature and also linked to time on the line. Taken together, our results suggest that elevated fight times and temperatures increase the physiological stress experienced by rod and reel-caught flapper skate. Efforts to reduce fight times and minimize heat exposure (including shading, irrigation and reduced handling time) should be beneficial for skate.

## Introduction

Catch and release angling of elasmobranchs is globally common (Freire *et al.,* 2020) and popular in UK waters (Jones *et al.,* 2021; Thomas *et al.,* 2023)*.* Sport fisheries have socio-economic benefits (Hyder *et al.,* 2020) and support scientific research in many systems (Brownscombe *et al.,* 2019). However, angling can also cause physical injury and physiological disturbances that affect behaviour (Knotek *et al.,* 2022), reproduction (Sutter *et al.,* 2012) and survival (Skomal*,* 2007*;*Mohan *et al.,* 2020)*.* These impacts are well studied in teleosts (Arlinghaus *et al.,* 2007*)*, but have received less attention in elasmobranchs (Horton *et al.,* 2023), with much existing research focused on the survival of sharks captured by commercial fisheries. Many taxa, especially batoids, remain understudied (Skomal and Mandelman, 2012)*.*

The capture and handling of elasmobranchs by various fishing methods can induce profound physiological disturbances (Hoffmayer and Parsons, 2001; Hyatt *et al.,* 2012; Skomal and Mandelman, 2012). Capture is usually associated with bursts of muscular activity and exhaustive anaerobic exercise. Anaerobic respiration is a normal physiological response to exercise demands such as burst swimming or excitation (Kieffer, 2000). However, when exercise demands are intense or prolonged, marked and potentially fatal acid–base, ionic, osmotic and fluid balance alterations can occur. Metabolic acidosis frequently occurs in captured elasmobranchs and is typically characterized by a decrease in blood pH and buffers, and an increase in blood lactate (Skomal and Mandelman, 2012). Lactate accumulation occurs as a result of anaerobic respiration and is accompanied by the generation of hydrogen ions and a lowering of the pH, with subsequent depletion of bicarbonate ions (Richards *et al.,* 2003). Respiratory acidosis occurs when ventilation is compromised, for instance due to mouth hooking or gill compression, leading to increased blood carbon dioxide levels and a decrease in blood pH (Frick *et al.,* 2012). Acidosis induced by capture may be primarily metabolic or respiratory in origin or a combination of both (Mandelman and Skomal, 2009; Frick *et al.,* 2012; Hyatt *et al.,* 2018). Alterations in other blood parameters, including increased glucose concentrations (due to activation of the glucocorticoid response) and changes in potassium and magnesium levels, have also been documented in elasmobranchs in response to capture stress (Cliff and Thurman, 1984; Moyes *et al.,* 2006; Cicia *et al.,* 2012).

Tolerance to capture and handling differs between species (Mandelman and Skomal, 2009; Hyatt *et al.,* 2012; Gallagher *et al.,* 2014). Benthic elasmobranchs are generally considered more tolerant to restraint than pelagic species (Naples *et al.,* 2012) due to their respiratory mode (buccal pumping versus ram ventilation) (Manire *et al.,* 2001) and differences in metabolic rate and aerobic scope (Molina *et al.,* 2020). However, responses vary even between closely related species (Ellis *et al.,* 2017; Knotek *et al.,* 2018) and with capture context (including capture method and duration), individual characteristics (including size and sex) and environmental conditions (especially temperature) (Danylchuk *et al.,* 2014; Ellis *et al.,* 2017). For example, longer fight times are commonly associated with metabolic acidosis (Skomal, 2007; Kneebone *et al.,* 2013; Mohan *et al.,* 2020). Warmer temperatures have also been linked to exacerbated physiological stress responses and increases in mortality (Cicia *et al.,* 2012; Hoffmayer *et al.,* 2012; Hyatt *et al.,* 2018). Understanding how and why these effects vary within and amongst systems is important for species’ conservation, especially for vulnerable species targeted by recreational angling (Arostegui *et al.,* 2021).

The flapper skate (*Dipturus intermedius*) is a critically endangered, benthic elasmobranch that was formerly distributed across northern Europe, but subsequently widely extirpated by commercial fisheries (Brander, 1981; Ellis *et al.,* 2024). However, the species remains locally abundant off the west coast of Scotland, where recreational catch-and-release angling records, and electronic tagging and tracking, supported the designation of the Loch Sunart to the Sound of Jura Marine Protected Area (LStSJ MPA) (Neat *et al.,* 2015; Dodd *et al.,* 2022). Management in the MPA restricts commercial fisheries but permits recreational angling. Angler-derived data, alongside electronic tagging and tracking, in collaboration with Scotland’s Nature Conservation Agency (NatureScot), has informed our understanding of the ecology and conservation of this species and supports ongoing monitoring (Thorburn *et al.,* 2021; Lavender et al., 2021a, 2021b). However, the impacts of angling on skate remain uncertain. Although recapture statistics suggest high survivorship (Régnier *et al.,* 2024), behavioural disturbances have also been documented (Lavender *et al.,* 2022a). Further research on the impacts of this practice, namely the nature and degree of the physiological stress response, is required to inform mitigation measures.

In this study, we document physiological responses of flapper skate to rod and reel capture, handling and the surgical implantation of acoustic tags. From 2018–20, we captured skate in the LStSJ MPA and measured blood parameters at two time points (immediately after capture and immediately prior to release) and recorded heart and respiratory rates during handling. We investigated relationships between blood parameters, heart/respiratory rates and aspects of the capture and handling process (such as fight time), individual characteristics (such as size) and environmental conditions (such as temperature) to examine whether captured skate exhibited metabolic and respiratory acidosis. We discuss the implications of our results for skate conservation.

## Materials and Methods

### Study site

Flapper skate were captured in the LStSJ MPA at favoured ‘angling marks’ (Fig. S1). The bathymetric environment spans shallow coastal waters alongside deep-water sections (up to 290 m in depth) (Howe *et al.,* 2014). Water temperatures vary from a winter minimum of ~6°C to a latesummer maximum of ~16°C. Over the summer, a thermocline 1–2°C in magnitude develops in the upper (<100 m) water layers. Air temperatures vary from ~−2 to 22°C. Semi-diurnal tides and seasonal wind variability dominate the current flow regime (Aleynik *et al.,* 2022).

### Data collection

#### Capture

Sixty-two skate captures were recorded (comprising 61 individuals, of which one individual was captured twice). Skate were caught from a charter angling vessel between August 2018 and March 2020 (Fig. S1, Table S1). Bottom temperatures (±0.1°C) were recorded using a Star Oddi milli-TD archival tag attached to the vessel’s anchor. Angling gear was standardized and followed typical angling practices (see Supporting Information §1.1). For all angling and veterinary equipment, see Table S2. Captured skate were brought onto the vessel either by sliding a sling under the skate or by using a gaff hooked through the leading edge of the wing (as per NatureScot, 2023). On the vessel, skate were placed onto a closed-cell foam mat, in ventral recumbency, shaded and supplied with seawater supplemented with medical oxygen via the spiracles (using a hose). Skate were sexed (by the presence or absence of claspers) and their total length (snout to tail tip) and disc width (wing tip to wing tip) were measured. Individual ‘health status’ was classified from physical examination and the presence/absence of injuries/infections (see Supporting Information §1.2). Healthy individuals (*n* = 55, including one recaptured individual) were blood sampled at two time points and surgically tagged with acoustic transmitters before release. Heart and respiratory rates were recorded throughout handling. Individuals with injury or infection (*n* = 6) were assessed and treated by a veterinarian but excluded from all analyses (see Supporting Information §1.2).

#### Blood sampling

Blood samples were taken from skate immediately after landing (‘blood sample one’: BS1) and immediately prior to release (‘blood sample two’: BS2). We obtained 51 samples at BS1 and 46 samples at BS2 from healthy individuals. (For two individuals, we obtained BS2 but not BS1.) Blood was sampled from the caudal vein (ventral coccygeal) using a 21G needle and syringe (see Supporting Information §1.3). Blood samples were considered predominantly venous; however, due to the anatomical proximity of the caudal vein and artery some samples may have been mixed (Mandelman and Skomal, 2009). Samples were immediately placed into a collection tube containing lithium heparin and inverted. Whole blood (95 μl) was pipetted into a CG4+ cartridge within 10 min of sampling (minimizing blood–air mixing) and analysed with the i-STAT handheld analyser for pH, carbon dioxide partial pressure (PCO_2_), oxygen partial pressure (PO_2_), bicarbonate and lactate. Glucose was measured within 10 min using an Accu-Check® mobile glucometer. Remaining blood samples were placed into an insulated cooler with ice packs. Plasma was separated by centrifugation within 10 h and stored, initially at −20°C (for ≤5 days) and subsequently at −80°C (until analysis). After defrosting at room temperature, plasma potassium (K) and magnesium (Mg) were measured using an AU480 chemistry analyser by the ion selective electrode method and colorimetry, respectively. We measured these parameters because of their elevation in response to capture in several elasmobranch species, and their ability to predict post-release survival in longline-captured blue sharks (*Prionace glauca*) (Cliff and Thurman, 1984; Moyes *et al.,* 2006; Cicia *et al.,* 2012). For each blood parameter, we successfully obtained 28–50 measurements at BS1 and 18–43 at BS2. Missing values were due to equipment failure and sample quality (clotted or insufficient volumes of blood).

#### Tagging

Forty-one skate were surgically tagged (between BS1 and BS2) with acoustic transmitters as part of a wider research project (Table S1). Skate were not tagged in rough seas or if available tags were not the appropriate size. Skate were placed in dorsal recumbency for tagging and oxygenated seawater was supplied via the mouth. A ventral mid-line incision (~3 cm) was made into the coelomic cavity after local anaesthesia of the incision site by infiltration with lidocaine. An Innovasea V16 or V13 tag was placed in the coelomic cavity and the incision closed in two layers (coelomic membranes and muscle followed by skin) with a monofilament absorbable suture (see Supporting Information §1.4 for additional details). Tagging lasted ~5–10 min.

#### Heart and respiratory rates

Throughout the capture process, respiratory and heart rates were recorded (for 30–60 s) at ~5-min intervals, from observation of buccal/spiracle movements and cardiac contractions. Heart rates were monitored via ultrasound with skate in dorsal recumbency (Table S3).

#### Ethics

Data collection was reviewed and approved by the Ethics Committees of the University of St Andrews (number SEC21024) and the Royal Zoological Society of Scotland. All regulated procedures involving animals were carried out under Home Office Project Licence number P05E95C50 according to The Animals (Scientific Procedures) Act 1986.

### Statistical modelling

#### Capture events

Data were analysed in R, version 4.2.3, using the stats, finalfit and mgcv packages (Wood, 2017; Harrison *et al.,* 2021; R Core Team, 2021). See Table S4 for a summary of analyses. Since fight time is known to influence physiological responses to capture, we first analysed the relationship between fight time, individual characteristics (sex and body size) and environmental variables (time of day, water temperature and depth), using a generalized linear model (GLM). This model is described in the Supporting Information §2. Here, we focus on the physiological analyses.

#### Blood parameters

We analysed physiological responses to capture, handling and tagging using blood parameter measurements for pH, PCO_2_, PO_2_, bicarbonate, lactate, glucose, potassium and magnesium. Prior to analysis, we applied temperature corrections to pH, PCO_2_ and PO_2_ to account for the discrepancy between ambient water temperature (assumed body temperature) and measurement temperatures, as the i-STAT warms samples to 37°C for measurement (see Supporting Information §3.1). Bicarbonate was calculated from temperature-corrected pH and PCO_2_ values (see Supporting Information §3.1). All samples that failed quality checks were excluded (see Supporting Information §3.2). Following a three-step workflow, we then investigated relationships between blood samples, individual characteristics and aspects of the capture process.

**Physiological state (Step 1).** We analysed the physiological state of individuals at BS1 and BS2 by visualizing distribution of values for each blood parameter and modelling values using GLMs. For BS1, we considered each blood parameter $(\mathrm{BS}{1}^{(j)})$in relation to sex (females, $\mathrm{se}{\mathrm{x}}_{\mathrm{F}}$, versus males, $\mathrm{se}{\mathrm{x}}_{\mathrm{M}}$), body size (total length, centimetres), bottom temperature (°C), the time (minutes) from hooking to the surface (i.e. fight time), the time from the surface to the blood sample and a factor distinguishing non-gaffed/gaffed ($\mathrm{gaf}{\mathrm{f}}_{\mathrm{N}}$/$\mathrm{gaf}{\mathrm{f}}_{\mathrm{Y}}$) individuals. An interaction was included between bottom temperature and fight time since exhaustive exercise is likely to have greater impacts in warmer, less oxygenated water. Each model took the form:


(1) \begin{align*}\mathrm{BS}{1}_i^{(j)}&\sim N\left({\mu}_i,{\sigma}^2\right) \\ \log \left({\mu}_i\right)&={\beta}_0+{\beta}_1\mathrm{se}{\mathrm{x}}_{{\mathrm{M}}_i}+{\beta}_2\mathrm{siz}{\mathrm{e}}_i+{\beta}_3\mathrm{tem}{\mathrm{perature}}_i\nonumber\\&\quad+{\beta}_4\mathrm{tim}{\mathrm{e}}_{\mathrm{hook}\to \mathrm{surfac}{\mathrm{e}}_i}\nonumber\\ &\quad+{\beta}_5\mathrm{temperatur}{\mathrm{e}}_i\mathrm{tim}{\mathrm{e}}_{\mathrm{hook}\to \mathrm{surfac}{\mathrm{e}}_i}\nonumber\\&\quad+{\beta}_6\mathrm{tim}{\mathrm{e}}_{\mathrm{surface}\to \mathrm{BS}{1}_i}+{\beta}_7\mathrm{gaf}{{\mathrm{f}}_{\mathrm{Y}}}_i\nonumber \end{align*}

where $i$ indexes observations. At BS2, we also exploited the fact that seven individuals were not tagged (due to tag availability or sea state) to investigate putative effects of tagging, by including a factor in the model that distinguished untagged from tagged individuals (${\mathrm{surgery}}_{\mathrm{Y}}$). BS2 models took the form:


(2) \begin{align*}\mathrm{BS}{2}_i^{(j)}&\sim N\left({\mu}_i,{\sigma}^2\right)\\ \log \left({\mu}_i\right)&={\beta}_0+{\beta}_1\mathrm{se}{\mathrm{x}}_{{\mathrm{M}}_i}+{\beta}_2\mathrm{siz}{\mathrm{e}}_i+{\beta}_3\mathrm{tem}{\mathrm{perature}}_i\nonumber\\&\quad+{\beta}_4\mathrm{tim}{\mathrm{e}}_{\mathrm{hook}\to \mathrm{surfac}{\mathrm{e}}_i}\nonumber\\&\quad+{\beta}_5\mathrm{temperatur}{\mathrm{e}}_i\mathrm{tim}{\mathrm{e}}_{\mathrm{hook}\to \mathrm{surfac}{\mathrm{e}}_i}\nonumber\\&\quad+{\beta}_6\mathrm{tim}{\mathrm{e}}_{\mathrm{surface}\to \mathrm{BS}{2}_i}+{\beta}_7\mathrm{gaf}{{\mathrm{f}}_{\mathrm{Y}_i}}+{\beta}_8\mathrm{surger}{\mathrm{y}}_{{\mathrm{Y}}_i}.\nonumber \end{align*}

See Supporting Information §3.3 for a justification of the model formulation.

**Physiological changes (Step 2).** We investigated the change in blood parameter values from BS1 to BS2, in three stages. First, we calculated and summarized the changes in blood parameters from BS1 to BS2. Second, for the subset of individuals with observations at both BS1 and BS2, we tested for significant differences between blood parameter values at BS1 and BS2 using percentile bootstrap tests for paired samples for (i) all individuals, (ii) tagged individuals and (iii) untagged individuals (see Supporting Information §3.4 for implementation details). For the subset of variables that changed significantly between BS1 and BS2, we modelled the magnitude of the changes in relation to explanatory variables by modifying Equation (2) as follows:


(3) \begin{align*} \Delta{\mathrm{BS}}_i^{(j)}&\sim N\left({\mu}_i,{\sigma}^2\right)\\{\mu}_i&={\beta}_0+{\beta}_1\mathrm{se}{\mathrm{x}}_{{\mathrm{M}}_i}+{\beta}_2\mathrm{siz}{\mathrm{e}}_i+{\beta}_3\mathrm{tem}{\mathrm{perature}}_i\nonumber\\&\quad+{\beta}_4\mathrm{tim}{\mathrm{e}}_{\mathrm{hook}\to \mathrm{surfac}{\mathrm{e}}_i}\nonumber\\&\quad+{\beta}_5\mathrm{temperatur}{\mathrm{e}}_i\mathrm{tim}{\mathrm{e}}_{\mathrm{hook}\to \mathrm{surfac}{\mathrm{e}}_i}\nonumber\\&\quad+{\beta}_6\mathrm{tim}{\mathrm{e}}_{\mathrm{surface}\to \mathrm{BS}{1}_i}+{\beta}_7\mathrm{tim}{\mathrm{e}}_{\mathrm{BS}1\to \mathrm{BS}{2}_i}+{\beta}_8\mathrm{gaf}{{\mathrm{f}}_{\mathrm{Y}_i}}\nonumber\\&\quad+{\beta}_9\mathrm{surger}{\mathrm{y}}_{{\mathrm{Y}}_i}\nonumber \end{align*}

where $\Delta{\mathrm{BS}}_i^{(j)}=\mathrm{BS}{2}_i^{(j)}-\mathrm{BS}{1}_i^{(j)}$; $\mathrm{tim}{\mathrm{e}}_{\mathrm{BS}1\to \mathrm{BS}2}$ denotes the time between blood samples; and other terms are as previously described.

**Synthesis (Step 3).** For the three GLMs, a bootstrapping approach was used to compare the effects of each explanatory variable on blood parameters. For each variable, we defined a standardized measure of effect size as the mean ratio of the response between the second and the first factor level or between the maximum and the minimum value for that variable estimated from 5000 bootstrap simulations, whilst holding other variables constant (see Supporting Information §3.5). Effect ratios were visualized for each blood parameter and explanatory variable to identify notable associations.

#### Heart and respiratory rates

Heart and respiratory rates were examined to investigate the influences of individual variation, environmental conditions, capture and handling. We modelled each $\mathrm{rat}{\mathrm{e}}^{(j)}$ as a function of sex, body size (total length), fight time, bottom temperature, time spent at the water surface before being pulled aboard and factors distinguishing gaffed/non-gaffed individuals and tagged/untagged individuals. To account for multiple (*n* = 1–10) observations during time on deck for each capture event, a generalized additive modelling framework with random effects smoothers ($s$) for individual and time on deck was used. Each model took the form:


(4) \begin{align*} \mathrm{rat}{\mathrm{e}}_{i,t}^{(j)}&\sim \mathrm{Negative}\ \mathrm{Binomial}\left({\theta}_{i,t}\right)\\{\theta}_{i,t}&={\beta}_0+{\beta}_1\mathrm{se}{\mathrm{x}}_{{\mathrm{M}}_i}+{\beta}_2\mathrm{siz}{\mathrm{e}}_i+{\mathrm{\beta}}_3\mathrm{tem}{\mathrm{perature}}_i\nonumber\\&\quad+{\beta}_4\mathrm{tim}{{\mathrm{e}}_{\mathrm{hook}\to \mathrm{surface}_i}}\nonumber\\&\quad+{\beta}_5\mathrm{temperatur}{\mathrm{e}}_i\mathrm{tim}{{\mathrm{e}}_{\mathrm{hook}\to \mathrm{surface}_i}}\nonumber\\&\quad+{\beta}_6t\mathrm{im}{\mathrm{e}}_{\mathrm{surface}\to \mathrm{dec}{\mathrm{k}}_i}+{\beta}_7\mathrm{gaf}{\mathrm{f}}_{{\mathrm{Y}}_i}+{\beta}_8\mathrm{surger}{{\mathrm{y}}_{\mathrm{Y}_i}}\nonumber\\&\quad+s\left(\mathrm{even}{\mathrm{t}}_i\right)+s\left(\mathrm{even}{\mathrm{t}}_i,\mathrm{tim}{\mathrm{e}}_{\mathrm{deck}\to \mathrm{observatio}{\mathrm{n}}_{i,t}}\right).\nonumber \end{align*}

See Supporting Information §4 for an alternative model formulation we considered that was less supported by the data.

## Results

### Health status

Of the 61 captured individuals, six (10%) had impaired health (Table S5). Two individuals had acute (hooked through body wall) or chronic (old gaffing wound) injuries. Three individuals had evidence of one or more abscesses. One skate had suspected coelomitis.

### Fight time

Fight times for healthy individuals ranged from 9 to 55 (median = 20) min. The model of fight time revealed that fight times were longer on average for larger individuals and marginally shorter in warm water (Fig. S2). However, there was substantial variability amongst individuals and coefficient estimates overlapped with zero (Table S6).

### Blood parameters

#### Blood sample one

For each blood parameter, we obtained 28–50 and 18–43 measurements for analysis at BS1 (post-capture) and BS2 (prior to release), respectively (Table S7). At BS1, most explanatory variables were associated with one or more blood parameters (Figs. 2 and S3–10, Table S8). In general, uncertainty was high and in most GLMs coefficient estimates overlapped with zero. However, the effect–ratio analysis revealed that most effects were not distributed uniformly around zero but broadly positive or negative.

Higher bottom temperatures were broadly associated with lower pH (Figs. 2a andS3) and bicarbonate (Figs. 2a andS6) and higher PCO_2_ (Figs. 2b andS4), lactate (Figs. 2e andS7), glucose (Figs. 2f andS8), potassium (Figs. 2g andS9) and magnesium (Figs. 2h andS10). For these blood parameters, the effect ratios were largely below/above one (Fig. 2), but uncertainty was high, and in the GLMs estimated coefficients were only statistically significant for potassium and magnesium (Table S8). However, the result for potassium was influenced by one individual with a high value.

Longer fight times were associated with lower pH and bicarbonate and higher PCO_2_, lactate, glucose, potassium and magnesium levels (Figs. 2 and S3–10). In the effect–ratio analysis, the differences in predicted blood parameter values between the lowest and highest fight times were broadly negative or positive (whilst holding other variables constant). However, the effect was only statistically significant in the GLM for potassium.

There was mixed evidence for an interaction between bottom temperature and fight time (Figs. 2 and S3–S10, Table S8). In general, we observed relatively lower values for pH and bicarbonate, and higher values for lactate and potassium, at jointly elevated temperatures and fight times (Fig. 2). For pH, bicarbonate and lactate, interaction coefficients were non-significant (Table S8) and the distributions of effect ratios for temperature (between short and long fight times) and fight time (between cool and warm temperatures) partially overlapped (Fig. 2). For potassium, the interaction coefficient was significant (Fig. S9, Table S8) and effect ratios were substantially different (albeit partially overlapping: Fig. 2).

There was a clear negative effect of time from the surface to BS1 on pH and bicarbonate (Fig. 2a and2d, Table S8). The effect of surface time on lactate was broadly positive (Fig. 2e). There were no clear effects of surface time on other blood parameters.

Gaffing was associated with somewhat lower bicarbonate values (Fig. 2d) and elevated lactate (Fig. 2e) and glucose (Fig. 2f) values. For bicarbonate and lactate, these effects were uncertain and not statistically significant, but for glucose the estimated coefficient and the distribution of effects were positive (Fig. 2e, Table S8).

There were limited effects of sex and size on blood parameters. There was some evidence for marginally lower pH and bicarbonate values and marginally elevated lactate and glucose values in males, but differences were uncertain and spanned zero (Fig. 2, Table S8). Smaller individuals were similarly associated with lower pH and bicarbonate and higher glucose and magnesium values, but this size effect was also uncertain (Table S8).

#### Blood sample two

Results from modelling blood parameters at BS2 were broadly consistent with those from BS1 (Figs. 3 and S11–S16, Table S9). In line with BS1, higher bottom temperatures and longer fight times were generally associated with lower values for pH and bicarbonate and higher values for PCO_2_, lactate and glucose (Figs. 3 and S11–16). In contrast to BS1, for pH, the temperature effect was significant in both the GLM (Table S9) and effect–ratio analysis (Fig. 3a), but for other variables the effects of temperature and fight time were principally apparent in the latter, as for BS1 (Fig. 3). Similarly, there was limited evidence for an interaction between temperature and fight time in the effect–ratio analysis for some blood parameters; namely, PCO_2_, bicarbonate and glucose (unlike BS1, where effects on pH, lactate, bicarbonate and potassium were clearest). Time from the surface to BS2 (which includes handling and tagging) was linked with lower bicarbonate values, as in BS1, but only in the effect–ratio analysis. In line with BS1, gaffing was associated with lower pH (but not bicarbonate) levels, and moderately higher PCO_2_ and lactate (but not glucose) levels in the effect–ratio analysis (Fig. 3). Like BS1, smaller individuals continued to exhibit broadly lower pH and bicarbonate values at BS2 (Fig. 3). There were no clear effects of sex or tagging on any blood parameter.

#### Changes in blood parameters during handling and tagging

**Figure 1:**
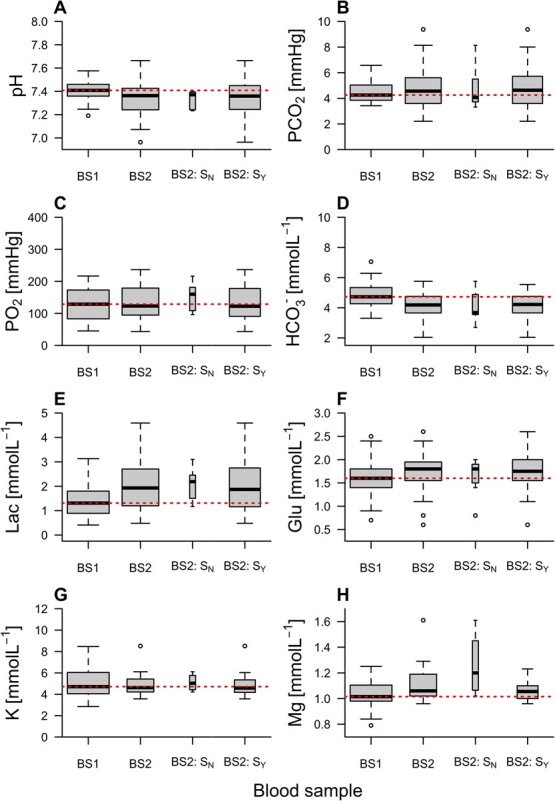
The distribution of blood parameter values in angled flapper skate at BS1 (post-capture) and BS2 (pre-release). BS2 is split by individuals that did ($\mathrm{Y}$) or did not ($\mathrm{N}$) undergo surgery ($\mathrm{S}$) during handling. On boxplots, the thick black line marks the median, the box edges mark the first (${Q}_1$) and third (${Q}_3$) quartiles and bar ends mark the range (excluding statistical outliers). The dashed line highlights the median at BS1. Hollow points mark statistical outliers (values $<{Q}_1 - 1.5\times \mathrm{IQR}$ or $>{Q}_3+1.5\times \mathrm{IQR}$, where $\mathrm{IQR}$ is the interquartile range). Box width is proportional to the number of observations.

Most blood parameters showed evidence of change between BS1 and BS2 (Figs. 1 and S17–S21, Tables S10–S11). Declines in pH and bicarbonate and increases in lactate and glucose from BS1 to BS2 were statistically significant (Table S10). The changes were broadly consistent between untagged/tagged individuals (Fig. 1, Table S10). The main exception to this was magnesium, which increased significantly in the small group of untagged individuals but not tagged individuals (Table S10).

**Figure 2:**
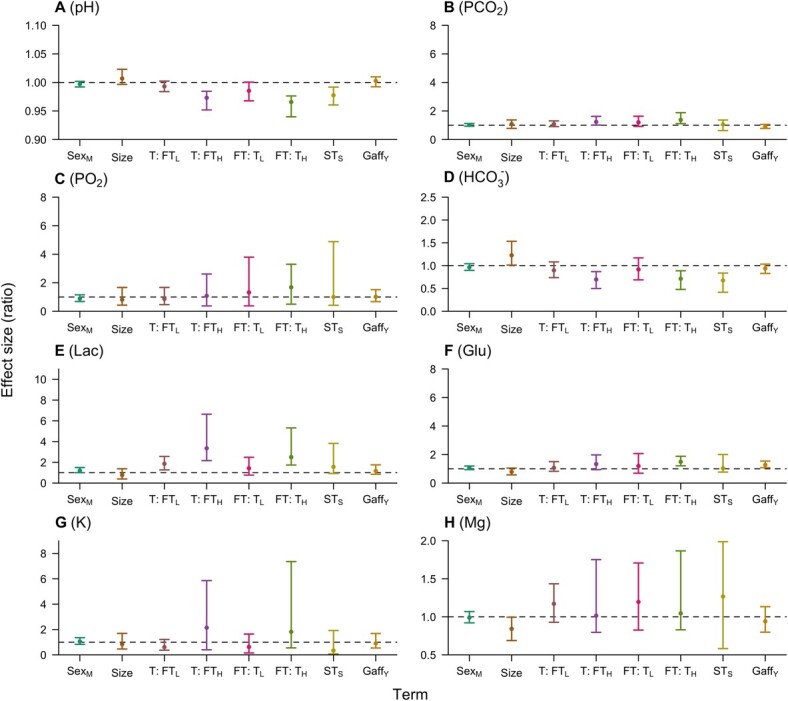
Effect ratios for (A) pH, (B) PCO_2_, (C) PO_2_, (D) bicarbonate, (E) lactate, (F) glucose, (G) potassium and (H) magnesium in angled flapper skate at BS1. In each panel, points show the mean effect size of specific explanatory variable ±95% confidence intervals (vertical bars). Effect ratios are defined as the ratio between simulated values of the blood parameter at the second, versus the first, factor level (for sex and gaffing), or the highest, versus the lowest, value (for continuous explanatory variables), whilst holding other variables constant. An effect $\mathrm{ratio}<1$ implies a decrease in blood parameter values; $\mathrm{ratio}=1$ (highlighted by the dashed horizontal line) implies no change; and $\mathrm{ratio}>1$ implies an increase. For example, values for $\mathrm{Se}{\mathrm{x}}_{\mathrm{M}}$ represent the proportional change in blood parameter values for males versus females, with other variables held constant at the first factor level or median. Subsequent labels are as follows: $\mathrm{Size}$ (total length), $\mathrm{T}$ (temperature), $\mathrm{FT}$ (fight time), $\mathrm{S}{\mathrm{T}}_{\mathrm{S}}$ (time from surface to blood sample) and $\mathrm{Gaf}{\mathrm{f}}_{\mathrm{Y}}$ (gaffed). Temperature and fight time effects are shown for the lowest (L) and highest (H) values of the other variable, given the interaction between these terms in the model.

**Figure 3:**
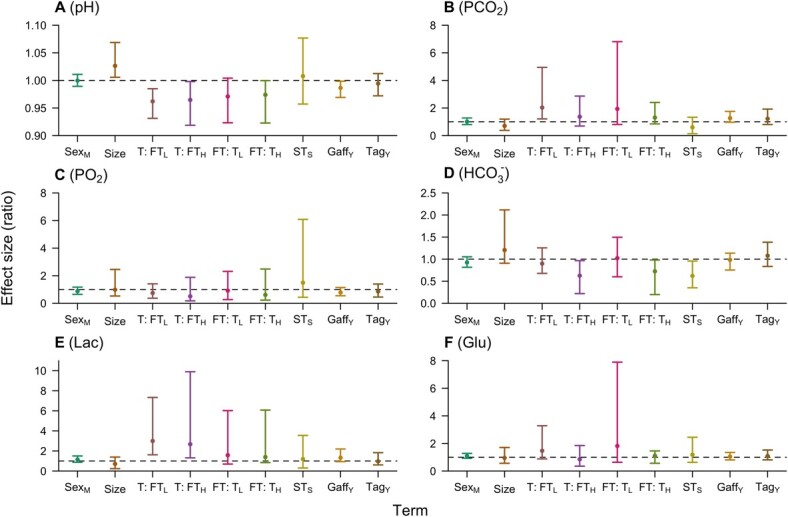
Effect ratios at BS2 for angled flapper skate, following Fig. 2. This analysis included the effect of tagging ($\mathrm{Ta}{\mathrm{g}}_{\mathrm{Y}}$). Surface time ($\mathrm{S}{\mathrm{T}}_{\mathrm{S}}$) is time from the surface to BS2 (and includes handling and tagging procedures). There were insufficient data to model potassium or magnesium at BS2.

**Figure 4:**
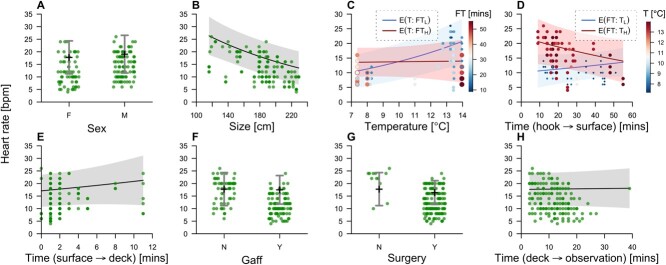
Heart rates of angled flapper skate in relation to (A) sex, (B) body size (total length), (C) bottom temperature, (D) fight time (E) surface time, (F) gaffing, (G) surgery and (H) deck time. Points mark observations for each capture event. In C and D these are coloured/sized by fight time and temperature, respectively. Black lines and surrounding bars/envelopes mark predictions and 95% confidence intervals from the model, with other variables held constant at the first factor level or median value, apart from in C and D in which the predicted effects of bottom temperature ($\mathrm{T}$) and fight time ($\mathrm{FT}$) are shown at both the lowest ($\mathrm{L}$) and highest ($\mathrm{H}$) value of the other variable, given the interaction term between these variables in the model. Confidence intervals include uncertainty in the effect of the explanatory variable and the mean.

In GLMs of the change in blood parameter values from BS1 to BS2, there was some evidence for effects of temperature and gaffing (Figs. S17–S20, Table S11). In the GLM, warmer temperatures were associated with greater reductions in pH (principally at shorter fight times: Fig. S17 and S21, Table S11) and increases in lactate (Fig. S19, Table S11), although the effect ratio of the latter was highly uncertain in line with large variation amongst capture events (Fig. S21). The GLM also linked gaffing with greater reductions in pH and glucose (Table S11), but simulated effect ratios were variable.

### Changes in heart and respiratory rates

There was a moderate, positive correlation between heart and respiratory rates in healthy individuals (Spearman’s rank correlation *S* = 194 086, $\rho =$ 0.56, *n*$=$ 135, $P\le$ 0.05). Heart rates varied from 4–26 (median = 12) beats per minute. Heart rates were significantly higher for smaller individuals and in warmer water (Fig. 4; Table S12). The temperature effect was principally apparent at shorter fight times; at longer fight times in warmer water, heart rates were generally lower, although the interaction was not statistically significant ($P=0.064$). Respiratory rates varied between 2 and 28 (median = 12) respirations per minute and were generally higher for smaller individuals (${\beta}_2=-0.05,P=0.058$) and lower for those that spent more time at the surface (${\beta}_6=-0.053,P=0.037$) (Figs. S23–4, Table S13). During handling, no consistent changes in heart or respiratory rates were observed (Fig. 4; Figs. S22 and S24). Total handling time on deck ranged from 9 to 31 (median = 20) min for healthy individuals.

## Discussion

This is the first study to document physiological responses to capture, handling and tagging in the critically endangered flapper skate. Skate generally experienced a mixed metabolic and respiratory acidosis characterized by decreases in blood pH and bicarbonate and increases in lactate, PCO_2_ and glucose. The degree of acidosis was greater with longer fight times and warmer sea temperatures, and worsened during time on deck. However, there was no evidence that capture responses differed between tagged and untagged individuals. Heart and respiratory rates were collectively associated with time on the line, temperature and body size but remained stable during time on deck. Collectively, these results suggest that capture and handling in flapper skate leads to physiological changes associated with the secondary stress response. Where angling for flapper skate is permitted, we recommend mitigation measures that minimize fight time, handling time and air/heat exposure. Measures include the use of appropriate gear, hook removal in water (where possible), and the provision of shade and irrigation. Guidelines produced in collaboration with anglers, and angler participation in monitoring schemes, may help to maintain best practices and support the contribution of angling to skate conservation (Lavender *et al.,* 2022b; NatureScot, 2023). Current management, which permits angling in some areas but prohibits it in others, is consistent with our results, given uncertainty in the duration, severity and long-term consequences of physiological disturbances. However, further research on the cumulative impacts of angling on survivorship is warranted, given the species’ conservation status. This study adds to the limited evidence base on batoid responses to capture and handling and calls for increased research on this understudied taxon.

### Blood parameters

#### Temperature

Warmer sea temperatures were associated with acidosis, as evidenced by increased lactate and reduced pH and bicarbonate at BS1 and BS2. In warmer water, greater changes in pH and lactate during time on deck (between BS1 and BS2) were also apparent. Warmer temperatures were additionally associated with elevated potassium and magnesium concentrations at BS1, but data variability limits interpretation of this result. As poikilotherms, the basal metabolic rate and oxygen consumption of skate increases with temperature; hence, in warmer water with less dissolved oxygen, skate undergoing exhaustive exercise reach aerobic capacity faster, resulting in a switch to anaerobic respiration, lactate production and a reduction in blood pH and bicarbonate (Butler and Taylor, 1975; Di Santo and Bennett, 2011). Increased physiological disturbance at higher temperatures in relation to angling has been demonstrated in multiple shark species (Hoffmayer *et al.,* 2012; Danylchuk *et al.,* 2014; Knotek *et al.,* 2022). Temperature change during capture (from water to air) can also worsen acidosis, as shown in little skate (*Leucoraja erinacea*) (Cicia *et al.,* 2012). In our study, we focused on the effect of water temperature and made efforts to minimize heat exposure during handling, including shade provision and seawater irrigation. However, elevated air temperatures may further worsen physiological changes in summer. Whilst the impact of physiological changes at elevated temperatures remain unclear for flapper skate, higher temperatures have been linked to longer recovery times in blacknose sharks (*Carcharhinus acronotus*) (Knotek *et al.,* 2022) and reduced survival of juvenile lemon sharks (*Negaprion brevirostris*) (Danylchuk *et al.,* 2014). Notwithstanding differences in species’ biology and capture contexts, this suggests the duration and consequences of physiological disturbance in flapper skate in response to increased temperatures warrant further research.

#### Fight time

Longer fight times were associated with metabolic and respiratory acidosis, as shown by broadly elevated lactate, glucose and PCO_2_ levels and lower pH and bicarbonate concentrations (especially at BS1). We anticipate that these effects will continue to worsen with fight times longer than observed here, such as prolonged recreational angling events, where fight times >120 min are known (G. Cole, unpublished data). In other systems, fight time has been linked to physiological disturbances in elasmobranchs caught in recreational (Skomal, 2007; Kneebone *et al.,* 2013; Mohan *et al.,* 2020) and commercial gear (Dapp *et al.,* 2016; Hyatt *et al.,* 2018; but see Shea *et al.,* 2022), where fight time may predict post-release mortality (Mohan *et al.,* 2020; Whitney *et al.,* 2021). Whilst behavioural analyses for flapper skate indicate recovery after a period of rest from the physiological disturbance associated with prolonged capture fights (up to 1 h in duration), minimizing fight times should help to reduce capture stress in this species (Lavender *et al.,* 2022a).

#### Surface and handling time

Skate became more acidotic with increasing time at the surface (from surface to BS1) and total handling time (from surface to BS2). During time on deck (from BS1 to BS2), pH and bicarbonate declined and lactate and glucose increased. There was some evidence for worsening respiratory acidosis, with limited increases in PCO_2_, during time on deck. Changes in blood parameters between BS1 and BS2 were associated with temperature and gaffing. Worsening acidosis here is likely due to the combined effects of air exposure, handling and prior exhaustive exercise (exacerbated at warmer temperatures). Air exposure has been demonstrated to have a profound negative effect on elasmobranchs (Cicia *et al.,* 2012; Heard *et al.,* 2014; Lambert *et al.,* 2018) with even brief periods of exposure (35 s) causing marked increases in PCO_2_ in blacktip sharks (*Carcharhinus limbatus*) (Weber *et al.,* 2021). Whilst we irrigated the gills with seawater and supplemented oxygen, some air exposure occurred as skate were brought on deck. During recreational angling events, such air exposure periods may be more prolonged, as gill irrigation is not commonly practised, though total handling time should be shorter in the absence of tagging and blood sampling. Blood biochemical alterations in elasmobranchs induced by exhaustive exercise may take hours to reach a peak (Cliff and Thurman, 1984; Richards *et al.,* 2003; Frick *et al.,* 2010) and 12–24 h to normalize (Kneebone *et al.,* 2013). In flapper skate, a recovery timescale of this magnitude is consistent with behavioural analyses demonstrating that skate typically rest for several hours following tagging and continue to show signatures of behavioural disturbance in the 12 h following release (Lavender *et al.,* 2022a). However, estimation of recovery time remains an important area for future work.

#### Gaffing

There was some evidence that gaffing was associated with acidosis, with somewhat elevated glucose (at BS1) and reduced pH (at BS2). Although angling guides typically advise against gaffing (Carlson *et al.,* 2019), it is still used as a method of boarding. Yet few studies have measured its physiological consequences (Otway, 2015) and the links between gaffing and physiological disturbance remain uncertain. For large animals (such as flapper skate), carefully placed gaffs may reduce handling times (NatureScot, 2023). However, gaffing causes physical trauma and can affect survival in elasmobranchs (Musyl and Gilman, 2019). The longer term consequences of gaffing, such as infection, the energetic cost of healing wounds, loss of function and fatal organ damage (for poorly placed gaffs) will not be reflected in acute changes in blood biochemistry but are likely to be important.

#### Tagging

Tagging did not appear to influence blood parameters or heart and respiratory rates. However, sample size was limited and any alterations in blood parameters due to surgery may take time to occur. Additionally, we lack data on healing time, post-operative discomfort and the incidence of complications such as infection. A greater understanding of the effects of tagging procedures on fish is needed to ensure that protocols are developed that benefit welfare and science (Clemens *et al.,* 2023). Unfortunately, few reports describe the effects of intra-coelomic tag placement and surgical wound healing in elasmobranchs, despite the prevalence of this practice. However, existing studies report minimal evidence of gross pathology (Haulsee *et al.,* 2016; Smukall *et al.,* 2019). We recaptured a mature female 340 days following tagging and observed complete external healing of the surgical incision. Whilst further research is required to elucidate the short- and long-term impacts of tagging, especially in long-lived species, these are encouraging findings given the importance of electronic tagging and tracking for skate conservation (Lavender *et al.,* 2023).

#### Size

Smaller skate were generally more acidotic, with lower pH and bicarbonate (at BS1 and BS2, respectively). Whilst our sample size was limited, elevated sensitivity of smaller elasmobranchs to capture stress in commercial trawl fisheries has been documented in skates (Depestele *et al.,* 2014; Knotek et al., 2020) and rays (Stobutzki *et al.,* 2002), as well as blue sharks caught in longlines (Diaz and Serafy, 2005; Coelho *et al.,* 2013) and recreational gear (Shea *et al.,* 2022). Possible explanations for these findings include increased susceptibility to trauma and fewer energy reserves (Knotek et al., 2020), increased susceptibility to temperature change (Spigarelli *et al.,* 1977; Prohaska *et al.,* 2021), a higher basal metabolic rate (per unit mass) and reduced buffering capacity in smaller individuals.

#### Sex

There was no effect of sex on blood parameters or heart and respiratory rates. Previous studies in trawl fisheries suggest that male mortality is elevated compared to females, which benefit from a thicker integument (Enever *et al.,* 2009; Mandelman *et al.,* 2013). This difference is less relevant in catch-and-release angling settings, which may explain the lack of sex difference in our study. However, sex-specific differences in capture responses to angling would benefit from further research, given the limited sample size in this study.

#### Physiological synthesis

Whilst baseline physiological values from flapper skate are lacking, comparison of our measurements with available estimates from other species suggests a mild to moderate degree of acidosis. In general, elasmobranch resting blood lactate concentrations are <1 mmoll^−1^ (Cicia *et al.,* 2012; Naples *et al.,* 2012; Speers-Roesch *et al.,* 2012a). This is broadly lower than values documented here, which averaged 1.31 and 1.93 mmoll^−1^ at BS1 and BS2 (range: 0.41–4.59 mmoll^−1^), especially in warmer temperatures and at longer fight times. Similarly, approximate baseline pH estimates at comparable temperatures range from 7.64 to 7.84 (Butler and Taylor, 1975; Cicia *et al.,* 2012; Frick *et al.,* 2012; Speers-Roesch *et al.,* 2012b), which broadly exceed the values we report (6.96–7.66), especially at BS2.

### Heart and respiratory rates

Heart and respiratory rates were collectively associated with body size, temperature and time on the line, but did not change during time on deck. In both cases, smaller individuals generally had higher rates. This size effect is expected and has been documented in elasmobranchs (Lyon, 1926; Dowd *et al.,* 2006). Heart and respiratory rates are also known to increase with temperature, in line with increases in metabolic rate and oxygen consumption (Butler and Taylor, 1975; Dowd *et al.,* 2006). Heart rate also increases moderately in elasmobranchs in response to exercise (Scharold *et al.,* 1989; Scharold and Gruber, 1991), although (in contrast to endothermic species) increases in cardiac output are modulated mainly by increases in stroke volume (Brill and Lai, 2015). In the model of heart rates, there was limited evidence for an interaction between fight time and temperature, with increasing fight times associated with slight increases in heart rates at cooler temperatures but decreases at warmer temperatures. A possible explanation for this result is that progressive hypoxia is occurring in warmer temperatures, as higher metabolic rates combined with exhaustive exercise and impaired ventilation in mouth-hooked skate result in oxygen demands exceeding supply. Hypoxia-induced bradycardia has been experimentally demonstrated in other elasmobranchs (Butler and Taylor, 1975; Speers-Roesch *et al.,* 2012a; Stensløkken *et al.,* 2004) and in dogfish was observed earlier (at higher critical oxygen tension) with increasing temperature (Butler and Taylor, 1975). In waters at the upper end of their thermal tolerance, elasmobranchs reach their maximum aerobic scope faster than in cooler waters (Farrell *et al.,* 2009). There was limited evidence for effects of temperature or fight time on respiration rates but the apparent decline in rates for individuals held at the surface for longer suggests a degree of recovery during this period. Collectively, these results suggest that skate may be exceeding their aerobic scope when exposed to long fight times in warmer water. Whilst oxygen supplementation has the potential to depress respiratory rates, we observed no change in rates during monitoring.

### Injuries

We documented injury or infection in 10% of individuals. Two individuals had injuries directly attributable to angling—one individual had been injured by a hook through the body wall that penetrated the coelom and another exhibited an unhealed wound consistent with previous gaffing. In other systems, hooking injuries are relatively common and can impact survival (Kneebone *et al.,* 2013; Danylchuk *et al.,* 2014; Cameron *et al.,* 2023), but their wider prevalence and consequences in flapper skate remain uncertain. Three skate had one or more abscesses and one had suspected coelomitis. Abscess formation has been reported in some captive elasmobranchs but not previously in free-ranging populations (Clarke *et al.,* 2013; Delaune and Anderson, 2020). It was not possible to determine the aetiology of abscess formation here, but penetrating injuries or impaired immunity due to other stressors are plausible explanations. Little is known about disease in wild elasmobranchs (Garner, 2013) and these results suggest further investigation into the incidence, aetiology and drivers of disease in flapper skate is warranted.

### Conservation implications

Physical trauma and physiological disturbance due to angling is linked with post-release mortality in elasmobranchs (Cameron *et al.,* 2023). Whilst data are limited and differences in capture contexts limit comparisons between systems, it is noteworthy that our lactate measurements (0.41–4.59 mmoll^−1^) lie broadly within the range tolerated by blue sharks on longlines (mean = 5.80 ± 2.96 [standard error] mmoll^−1^) (Moyes *et al.,* 2006) and in general are lower than the values associated with mortality in other species (Dapp *et al.,* 2016; Mohan *et al.,* 2020; Whitney *et al.,* 2021). In line with this result, short-term survival for all skate tagged in this study was inferred from acoustic data (Thorburn *et al.,* 2022). Whilst flapper skate caught in other circumstances may experience more significant physiological disturbances, angler mark-recapture data demonstrate that flapper skate in the MPA can survive multiple capture events, and annual survival rates in the MPA are high (~90%) (Régnier *et al.,* 2024). That being said, other effects of fishing such as premature abortion and reduced maternal/offspring fitness are reported in batoids (Guida *et al.,* 2017; Adams *et al.,* 2018; Wosnick *et al.,* 2019). Egg release associated with capture has been recorded in flapper skate (Benjamins *et al.,* 2021), but sub-lethal effects on maternal/offspring health and reproductive capacity are unknown. The circumstances under which capture-induced physical and physiological disturbance reduce survival probability, the frequency and magnitude of these occurrences and their population-level consequences, therefore remain knowledge gaps for skate conservation in areas where catch-and-release angling occurs.

## Supplementary Material

Web_Material_coae077

## Data Availability

Skate data and code are available on GitHub via https://github.com/edwardlavender/rzss-flapper and are archived on Zenodo (DOI: 10.5281/zenodo.11213308).
