# Supplementary material for: Physiological responses to capture, handling and tagging in the critically endangered flapper skate (Dipturus intermedius)
Source: Conserv Physiol. 2024 Nov 28;12(1):coae077. doi: 10.1093/conphys/coae077 (PMC11604123; doi:10.1093/conphys/coae077)
Supplement: Web_Material_coae077 [file web_material_coae077.zip › supp-materials.pdf]

Supporting information

Supporting materials

Contents

**Section**	**Pages**
Supporting information	1–12
Supporting figures	13–38
Supporting tables	39–59

Supporting information

1. Skate capture and handling

1.1. Capture

We captured skate using 50–80 Ib class rods, each with a lever drag multiplier reel, a braided main line, a 3 m 250 Ib rubbing length and a standard lead weight (12 oz) attached via a sliding boom. The tackle terminated in a 10/0 O'Shaughnessy hook with the barb removed. Whole Atlantic mackerel (*Scomber scombrus*) were used as bait.

On the vessel, individuals were shaded and provided with seawater supplemented with medical oxygen at 0.5 $Lmin^{-1}$. For large individuals (⪆ 120 cm in length), an additional hose pumping seawater without supplemental oxygen was used to provide sufficient water for respiration. As part of ongoing monitoring, individuals were measured (with a tape measure over the dorsal surface of the body), tagged with a miniature Passive Integrated Transponder tag (if necessary) and photographed. These data are recorded for entry into the Skatespotter database (https://skatespotter.sams.ac.uk/).

1.2. Health assessment

Skate were classified in one of two categories: 'healthy' or 'impaired health'. They were determined as healthy upon visual examination by a veterinarian if free of signs of significant injury or infection (including hooking injuries, suspected fractures, jaw damage, grossly observable abscesses or poorly healing wounds). 'Significant injury' was classified as injury

that might interfere with function (as is the case for injuries of the jaw or large muscle defects). Animals with superficial abrasions or injuries that appeared to be healing well were classified as healthy.

We focused the analyses in this paper exclusively on healthy individuals, given the limited number of individuals with impaired health and potential differences in the responses of such individuals to capture and handling. The wider prevalence of injuries/infections and the extent to which affected individuals experience more severe capture-related effects are important knowledge gaps for future work. At this stage, given limited data, we can say that the distribution of blood parameter values and heart/respiratory rates for unhealthy individuals overlapped with the distribution of values for healthy individuals (Table S2). However, we leave comprehensive analysis of the responses of healthy versus unhealthy individuals to capture and angling to future studies with larger sample sizes.

1.3.Blood samples

Two blood samples were taken from skate (denoted BS1 and BS2). The total volume of blood sampled (at both BS1 and BS2) was less than 0.5 % of individual body weight with a maximal total volume of 5 ml. Body weight was estimated from an angler length–weight chart (Holt, 2005).

1.4.Surgery

Intracoelomic tag implantation was performed as aseptically as possible. Surgical instruments were cleaned after each procedure, soaked in instrument sterilizing fluid according to manufacturer instructions and then rinsed thoroughly with sterile water prior to use. Similarly, acoustic tags were soaked in instrument sterilant after cleaning and removal of all stickers. Tags were thoroughly rinsed with sterile water prior to insertion in the celomic cavity. Sterile saline was used to irrigate the skin prior to surgery and sterile surgical gloves were worn throughout the procedure. Owing to the difficulty of safely anaesthetising large skate on a fishing vessel, a local anaesthetic block was used to prevent nociception at the incision site. Lidocaine 2% solution (ensuring total dose <1mgkg^{-1}) was injected subcutaneously along the incision line prior to surgery.

2. Capture fights

Fight time was defined as the duration from time on hook to time at the surface. In the analysis of fight time, we only considered individuals that were not captured whilst another individual was being processed since in these situations hooked individuals were only pulled to the surface after the individual on deck was nearly ready to be released. Following Lavender *et al.* (2022), we modelled fight time in relation to the following variables:

- **Sex ($\mathbf{sex_M}$).** A factor distinguishing males from females was included in the model.
- **Body size ($\mathbf{size}$).** Skate dorsal surface area (m^2) was included in the model as a metric of body size and as part of a term to account for the intertwined effects of body shape and water currents. For this analysis, following Lavender *et al.* (2022), individual surface area was approximated by treating each individual's disc width (cm) as the diagonal of a square, with area (m^2) given as ½(disc width/100)^2. The expectation was that larger skate should have longer fight times. Yet since skate are typically caught in the mouth and thus pulled up in a vertical position during capture, the influence of body size was expected to be greater for larger skate in line with the strength of water currents. For this reason, body size was included via an interaction with the current speed term in the model (see below).
- **Current speeds ($\mathbf{current}$).** For each capture event, the mean current speed in the water column was included to account for the influence of water currents on fight time. Current speeds were calculated from current velocity vectors extracted from the West Scotland Coastal Ocean Modelling System (Aleynik et al., 2016) via the `fvcom.tbx` package (Lavender, 2020) in R (R Core Team, 2023). This modelling system resolves hourly hydrodynamic conditions across an unstructured, triangular mesh that is organised into 11 vertically spaced, terrain-following Sigma layers (the 1st , 10th and 11th layers lie at the surface, seabed and below the seabed, respectively). Current velocity vectors are resolved at mesh centroids (elements). For each capture event, the current speed at each layer was calculated from current velocity vectors resolved in the nearest element and hour to the capture event (nearest neighbour interpolation). The ten current speed predictions were averaged to provide a metric of current speed broadly applicable throughout the capture fight. The influence of this metric was expected to be proportional to individual surface area and thus included via an interaction with body size in the model (see above).

- **Sun angle ($\mathbf{sun}$).** Sun angle above the horizon (°) at the onset of each capture event was calculated as a metric of 'biological time of day' using the `suncalc` package (Thieurmel & Elmarhraoui, 2019).
- **Temperature ($\mathbf{temperature}$).** Bottom temperature (°C), as recorded by the archival tag on the vessel's anchor at the onset of each capture event (specifically for the time interval nearest to the start of the capture event, given a recording resolution of two minutes), was included to capture variation in thermal performance and/or associated seasonality in activity levels.
- **Depth ($\mathbf{depth}$).** The depth (m) of the seabed in capture locations was extracted from 1 arc-second bathymetry data from Digimap. (Higher resolution data do not span the whole of the study area.)

We formulated the model as a generalised linear model (GLM) of the form:

$$\text{fight time}_i \sim N(\mu_i, \sigma^2) \tag{1}$$

$$\log(\mu_i) = \beta_0 + \beta_1 \text{sex}_{\text{M}_i} + \beta_2 \text{size}_i + \beta_3 \text{current}_i + \beta_4 \text{size}_i \text{current}_i + \beta_5 \text{sun}_i + \beta_6 \text{temperature}_i + \beta_7 \text{depth}_i,$$

where i indexes capture events.

In other settings, we recognise that additional variables, including previous capture history, environmental conditions such as dissolved oxygen levels, seabed sediment type and suspended sediment loads, and angler variables, such as experience and energy levels, can influence fight time (Mullen et al., 2020). In this study, the data required to account directly for these effects was insufficient. However, as argued by Lavender et al. (2022), their influence is expected to be limited.

For this GLM, and the ones described below, model fitting, prediction and diagnostic checks were implemented using standard functions in the R `stats` package (R Core Team, 2023). Statistical significance was defined based on the $p = 0.05$ threshold.

3. Blood parameter models

3.1. Blood parameter corrections

We measured pH, PCO_2, PO_2, and lactate using an i-STAT hand-held analyser (Gallagher et al., 2010). This machine warms blood samples (at ambient water temperature) to 37 °C for analysis. Post-hoc temperature corrections (for pH, PCO_2 and PO_2) were used to calculate values at ambient water temperature (approximated to skate body temperature) (Gallagher et al., 2010; Mandelman & Skomal, 2009). We corrected the measured (M) values for these parameters (at BS1 and BS2) using bottom sea temperature measurements (T, °C) from the archival tag on the vessel's anchor, for each capture event (i), as described below.

pH was corrected following Mandelman and Skomal (2009) according to the equation:

$$\mathrm{pH}_i = M_i - 0.011 \times (T_i - 37). \tag{2}$$

The constant 0.011 is derived from experimental studies of dogfish (*Scyliorhinus sp.*) (Heisler, 1988; Heisler et al., 1980; Heisler & Neumann, 1980).

PCO_2 (mmHg) was corrected following Mandelman and Skomal (2009) as determined by Nunn et al (1965), according to the equation:

$$\mathrm{PCO}_{2_i} = M_i \times 10^{-0.019(37-T_i)}. \tag{3}$$

PO_2 (mmHg) was corrected, as in Naples *et al.* (2012), according to the equation:

$$\mathrm{PO}_{2_i} = M_i \times 10^{-0.0058(37-T_i)}. \tag{4}$$

Using the temperature-corrected values for pH and PCO_2, we also calculated bicarbonate (HCO_3^-, mmolL^{-1}) from the Henderson-Hasselbalch equation (Keller et al., 2012):

$$\mathrm{HCO}^-_{3_i} = \alpha\mathrm{CO}_{2_i} \times \mathrm{PCO}_{2_i} \times 10^{(\mathrm{pH}_i - \mathrm{pK}_i)}, \tag{5}$$

where $\alpha\mathrm{CO}_2$ (mmolL^{-1}torr^{-1}) is the solubility of CO_2 in plasma and apparent pK is the carbonic acid dissociation constant. These terms are species-specific and temperature-dependent. As values for flapper skate are not available, we derived them using available formulas for dogfish (Boutilier et al., 1984). The term $\alpha\mathrm{CO}_2$ was defined according to the equation:

$$\alpha\mathrm{CO}_{2_i} = 0.1131 - 1.3847 \times 10^{-2}(T_i) + 1.4995 \times 10^{-3}(T_i^2) - 8.8008 \times 10^{-5}(T_i^3) + 2.4998 \times 10^{-6}(T_i^4) - 2.7369 \times 10^{-8}(T_i^5). \tag{6}$$

The term pK was defined as:

$$\mathrm{pK}_i = 6.4996 + \log(T_i)(0.3648 - 0.0521 \times \mathrm{pH}_i) - 0.0353 \times \mathrm{pH}_i - 0.0074 \times T_i, \tag{7}$$

where pH is temperature-corrected (Boutilier et al., 1984).

3.2. Quality checks

All blood parameter values were inspected for measurement errors and two observations were excluded from analyses for this reason (Table S3). For potassium, we also excluded samples that showed obvious evidence of haemolysis (red or dark pink plasma), which causes false increases in potassium values. Samples with very mild haemolysis (slight pinking of plasma) were included in analysis.

3.3. Physiological state

In the models of blood parameters, we considered each parameter in relation to sex, body size, fight time, handling time at the surface, bottom temperature and whether or not the individual was gaffed and tagged, if applicable (equations 1–2 in the Main Text). We specifically distinguished between fight time and time to the blood sample because the experience of skate during capture fights differs from the experience of skate during handling. We note that 'surface time to BS1 or BS2' includes time spent in the water at the surface as well as time on the vessel's deck when the skate was supplied with water via hoses but, given the volume of data available for blood parameter models, we did not distinguish between these types of 'surface time'. At BS2, we also exploited the fact that a small number of individuals were not tagged (N = 7) to investigate putative effects of tagging. Given available data, the purpose of this analysis was not to estimate precise coefficients for the effect of tagging but to flag any major differences in blood parameter values between untagged and tagged individuals.

We recognise that other variables, such as dissolved oxygen levels, thermocline strength and air temperature, are likely to affect physiological responses to capture (Cicia et al., 2012). In this study, the data required to evaluate the effects of these variables was unavailable. For example, while air temperature records are available for the study site, the ambient temperature on the deck of a vessel may differ from the study site at large. However, bottom temperatures correlate with air temperatures (even though the latter show more variability), as well as other hydrodynamic variables, such as dissolved oxygen levels. Furthermore, during handling on deck, the provision of shading and oxygenated seawater was designed to help maintain skate at ambient seawater temperatures. Coupled with the (large) size of captured skate, which ranged from 112–229 (median = 183) cm in length, these provisions should have minimised

the effects of temperature change (especially the effects of warmer air temperatures) during time on deck.

3.4. Blood parameter changes

We tested whether the change in blood parameter values from BS1 to BS2 for each blood parameter was statistically significant using percentile bootstrap paired-sample tests. In each case, we compared paired samples for three groups: (a) all individuals, (b) untagged individuals and (c) tagged individuals. Our expectation for this analysis was that pH and bicarbonate would decrease, while PCO_2, lactate, glucose, magnesium and potassium would increase (Cliff & Thurman, 1984; Heard et al., 2014; Moyes et al., 2006; Weber et al., 2021) . For PO_2 we did not have an *a priori* expectation for the direction of change; for example, while PO_2 has been shown to decline during handling in other studies (Weber et al., 2021), we anticipated that the provision of oxygenated seawater during handling could mitigate this effect (Weber et al., 2021). For each blood parameter and group, using the data from individuals for which values for BS1 and BS2 were successfully recorded, we used 1,000 bootstrap samples to estimate the mean difference and the associated *p*-value. This approach was implemented using the `boot.paired.per` function in the `wBoot` package (Weiss, 2016). *p*-values were corrected using the Bonferroni correction for multiple comparisons (separately for each group) via the `p.adjust` function in the `stats` package (R Core Team, 2023).

For the subset of variables that showed significant changes between BS1 and BS2 (pH, bicarbonate, lactate and glucose), we explicitly investigated whether the magnitude of the changes from BS1 and BS2 could be explained by individual characteristics, environmental conditions and aspects of the capture and handling process (as represented in models of BS1 and BS2). For example, we hypothesised that individuals with longer fight times would show greater declines in pH and bicarbonate and increases in lactate and glucose between BS1 and BS2 during time on deck. This model is described by equation (3) in the Main Text.

3.5. Synthesis

Using the GLMs of blood parameter values at BS1, BS2 and the change from BS1 to BS2 (equations 1–3 in the Main Text), we estimated standardised effect ratios from 5,000 bootstrap

simulations to facilitate the identification of associations between blood parameters and explanatory variables. Bootstrapping was implemented using the `finalfit` package (Harrison et al., 2021). For each GLM and blood parameter, effect ratios were computed from the distribution of ratios between simulated values of each blood parameter for the second versus first factor level or between the maximum versus minimum values of the explanatory variable, while holding other variables constant. We used this approach, rather than simply comparing model coefficients, because coefficients are estimated on the scale of the link function, which makes their biological significance in terms of the degree of change in blood parameter values difficult to interpret directly, unlike effect ratios. For the analysis, we considered the following effects:

- $\mathbf{Sex_M}$**.** The effect of being 'male' versus 'female'.
- **Size.** The effect of body size (total length) between the largest (229 cm) and smallest (116 cm) sampled sizes.
- $\mathbf{T{:}\,FT_L}$. The effect of the bottom temperature (T) between the warmest bottom temperatures (13.96 °C) versus the coolest bottom temperatures (7.20 °C), holding fight time (FT) at its lowest (L) value (8 minutes).
- $\mathbf{T{:}\,FT_H}$. The effect of bottom temperature, as above, holding fight time at its highest (H) value (55 minutes).
- $\mathbf{FT{:}\,T_L}$. The effect of fight time between the longest (55 minutes) and shortest (8 minutes) fights, holding bottom temperature (T) at its lowest value (7.20 °C).
- $\mathbf{FT{:}\,T_H}$. The effect of fight time, as above, holding bottom temperature at its highest value (13.96 °C).
- $\mathbf{ST_S}$**.** The effect of time from surfacing to BS1 (or BS2) between the maximum and minimum durations (36 versus 3 minutes for BS1 and 37 versus 8 minutes for BS2). Note that the individual with the longest surface time to BS1 (36 minutes) was not sampled at BS2, hence the small difference between these ranges.
- $\mathbf{ST_\Delta}$**.** The effect of time from BS1 to BS2 between the minimum and maximum durations (3 and 27 minutes, respectively). This effect was only investigated for models of the change in blood parameters from BS1 to BS2.
- $\mathbf{Gaff_Y}$**.** The effect of gaffing between gaffed versus non-gaffed individuals.
- $\mathbf{Tag_Y}$**.** The effect of being tagged at BS2. This effect was only investigated for models of BS2 and the change in blood parameters from BS1 to BS2.

For bottom temperature and fight time, simulations were implemented for both minimum and maximum values of the other variable, given the interaction between these terms in models. In other cases, simulations were implemented with the following constants: sex = 'F'; the median body size (total length: 183 cm); the median bottom temperature (13.24 °C); the median fight time (20 minutes); the median surface time until the blood sample (8 and 17 minutes for BS1 and BS2 respectively); the median time between blood samples, if applicable (10 minutes); gaff = 'N'; and tag = 'N' (if applicable). Note that while the effect ratios for each term indicate the relative statistical influence of each explanatory variable on each blood parameter across the range of values of each variable observed in this study, ratios for different explanatory variables are not directly comparable due to differences in the variability of different explanatory variables.

4. Heart and respiratory rates

Heart and respiratory rates were modelled in a generalised additive modelling (GAM) framework via the `mgcv` package in R (Wood, 2017). A random effects model formulation was used to account for repeated observations on deck during each capture event, with random effects for capture event and time on deck (equation (4) Main Text). Given the resolution of available data, we did not investigate the effects of the timing of blood samples on heart and respiratory rates, although variation in the experience skate during handing should be partially captured through the random effects terms.

One potential issue with our model formulation is that the effects of size and the random intercept term may have been conflated because 78 % of events were uniquely defined by the size of the individual captured. For this reason, we also fitted an alternative model replacing the random effects terms with an interaction between body size and time on deck, according to the equation:

$$
\begin{gathered}
\text{rate}_{i,t}^{(j)} \sim \text{Negative Binomial}(\theta_{i,t}) \\
\theta_{i,t} = \beta_0 + \beta_1 \text{sex}_{\text{M}_i} + \beta_2 \text{temperature}_i + \beta_3 \text{time}_{\text{hook}\to\text{surface}_i} + \\
\beta_4 t\text{emperature}_i \text{time}_{\text{hook}\to\text{surface}_i} + \beta_5 \text{time}_{\text{surface}\to\text{deck}_i} + \beta_6 \text{gaff}_{\text{Y}_i} + \\
\beta_7 \text{surgery}_{\text{Y}_i} + \text{te}\left(\text{size}_i, \text{time}_{\text{deck}\to\text{observation}_{i,t}}\right),
\end{gathered}
\tag{8}
$$

where te is a tensor product interaction. We compared our initial model (described in the Main Text) to this model using Akaike's Information Criterion (AIC). For both heart and respiratory

rates, our initial model was favoured (ΔAIC was 5.3 and 108.7 respectively) and this alternative model is not discussed further.

References

Aleynik, D., Dale, A. C., Porter, M., & Davidson, K. (2016). A high resolution hydrodynamic model system suitable for novel harmful algal bloom modelling in areas of complex coastline and topography. *Harmful Algae*, *53*, 102–117. https://doi.org/10.1016/j.hal.2015.11.012

Boutilier, R. G., Heming, T. A., & Iwama, G. K. (1984). *Appendix: Physicochemical Parameters for use in Fish Respiratory Physiology* (Vol. 10, pp. 403–430). Academic Press. https://doi.org/10.1016/S1546-5098(08)60323-4

Cicia, A. M., Schlenker, L. S., Sulikowski, J. A., & Mandelman, J. W. (2012). Seasonal variations in the physiological stress response to discrete bouts of aerial exposure in the little skate, Leucoraja erinacea. *Comparative Biochemistry and Physiology - A Molecular and Integrative Physiology*, *162*(2), 130–138. https://doi.org/10.1016/j.cbpa.2011.06.003

Cliff, G., & Thurman, G. D. (1984). Pathological and physiological effects of stress during capture and transport in the juvenile dusky shark, Carcharhinus obscurus. *Comparative Biochemistry and Physiology Part A: Physiology*, *78*(1), 167–173. https://doi.org/10.1016/0300-9629(84)90111-7

Gallagher, A. J., Frick, L. H., Bushnell, P. G., Brill, R. W., & Mandelman, J. W. (2010). Blood Gas, Oxygen Saturation, pH, and Lactate Values in Elasmobranch Blood Measured with a Commercially Available Portable Clinical Analyzer and Standard Laboratory Instruments. *Journal of Aquatic Animal Health*, *22*(4), 229–234. https://doi.org/10.1577/H10-012.1

Harrison, E., Drake, T., & Ots, R. (2021). finalfit: Quickly Create Elegant Regression Results Tables and Plots when Modelling. *R package version 1.0.4*. https://github.com/ewenharrison/finalfit

Heard, M., Van Rijn, J. A., Reina, R. D., & Huveneers, C. (2014). Impacts of crowding, trawl duration and air exposure on the physiology of stingarees (family: Urolophidae). *Conservation Physiology*, *2*(1). https://doi.org/10.1093/conphys/cou040

Heisler, N. (1988). Acid-Base Regulation. In T. Shuttleworth (Ed.), *Physiology of Elasmobranch Fishes* (pp. 215–248). Springer.

Heisler, N., & Neumann, P. (1980). The Role of Physico-Chemical Buffering and of Bicarbonate Transfer Processes in Intracellular pH Regulation in Response to Changes of Temperature in the Larger Spotted Dogfish *(Scyliorhinus Stellaris)*. *Journal of Experimental Biology*, *85*(1), 99–110. https://doi.org/10.1242/jeb.85.1.99

Heisler, N., Neumann, P., & Holeton, G. F. (1980). Mechanisms of Acid-Base Adjustment in Dogfish (Scyliorhinus Stellaris) Subjected to Long-Term Temperature Acclimation. *Journal of Experimental Biology*, *85*(1), 89–98. https://doi.org/10.1242/jeb.85.1.89

Holt, D. (2005). Common skate tagging programme. http://www.catchalot.co.uk/tagging/skate.htm

Keller, K. A., Innis, C. J., Tlusty, M. F., Kennedy, A. E., Bean, S. B., Cavin, J. M., & Merigo, C. (2012). Metabolic and respiratory derangements associated with death in cold-stunned Kemp's ridley turtles (Lepidochelys kempii): 32 cases (2005–2009). *Journal of the American Veterinary Medical Association*, *240*(3), 317–323. https://doi.org/10.2460/javma.240.3.317

Lavender E. (2020). fvcom.tbx: An R toolbox for the unstructured grid Finite Volume Community Ocean Model (FVCOM). *R package version 0.1.0.* (b; p. b). http://github.com/edwardlavender/fvcom.tbx

Lavender, E., Aleynik, D., Dodd, J., Illian, J., James, M., Wright, P. J., Smout, S., & Thorburn, J. (2022). Behavioural Responses of a Large, Benthic Elasmobranch to Catch-and-Release Angling. *Frontiers in Marine Science*, *9*, 864344. https://doi.org/10.3389/fmars.2022.864344

Mandelman, J. W., & Skomal, G. B. (2009). Differential sensitivity to capture stress assessed by blood acid–base status in five carcharhinid sharks. *Journal of Comparative Physiology B*, *179*(3), 267–277. https://doi.org/10.1007/s00360-008-0306-4

Moyes, C. D., Fragoso, N., Musyl, M. K., & Brill, R. W. (2006). Predicting Postrelease Survival in Large Pelagic Fish. *Transactions of the American Fisheries Society*, *135*(5), 1389–1397. https://doi.org/10.1577/T05-224.1

Mullen, E. J., Schoen, A. N., Hauger, M. D., Murray, L., & Anderson, W. G. (2020). Angler experience and seasonal effects on the response of the lake sturgeon to catch-and-release angling. *Transactions of the American Fisheries Society*, *149*(6), 709–720. https://doi.org/10.1002/tafs.10266

Naples, L. M., Mylniczenko, N. D., Zachariah, T. T., Wilborn, R. E., & Young, F. A. (2012). Evaluation of critical care blood analytes assessed with a point-of-care portable blood analyzer in wild and aquarium-housed elasmobranchs and the influence of phlebotomy

site on results. *Journal of the American Veterinary Medical Association*, *241*(1), 117–125. https://doi.org/10.2460/javma.241.1.117

Nunn, J. F., Bergman, N. A., Bunatyan, A., & Coleman, A. J. (1965). Temperature coefficients for Pco2 and Po2 of blood in vitro. *Journal of Applied Physiology*, *20*(1), 23–26. https://doi.org/10.1152/jappl.1965.20.1.23

R Core Team. (2023). *R: A language and environment for statistical computing.* https://www.R-project.org/

SAMS, & NatureScot. (n.d.). *SkateSpotter*. Retrieved June 3, 2024, from https://skatespotter.sams.ac.uk/

Thieurmel, B., & Elmarhraoui, A. (2019). suncalc: Compute Sun Position, Sunlight Phases, Moon Position and Lunar Phase. *R package version 0.5.1*. https://github.com/datastorm-open/suncalc

Weber, D. N., Janech, M. G., Burnett, L. E., Sancho, G., & Frazier, B. S. (2021). Insights into the origin and magnitude of capture and handling-related stress in a coastal elasmobranch *Carcharhinus limbatus*. *ICES Journal of Marine Science*, *78*(3), 910–921. https://doi.org/10.1093/icesjms/fsaa223

Weiss, N. A. (2016). wBoot: Bootstrap Methods. *R package version 1.0.3*. http://cran.nexr.com/web/packages/wBoot

Wood, S. N. (2017). *Generalized Additive Models: An Introduction with R*. Chapman and Hall/CRC.

Supporting figures

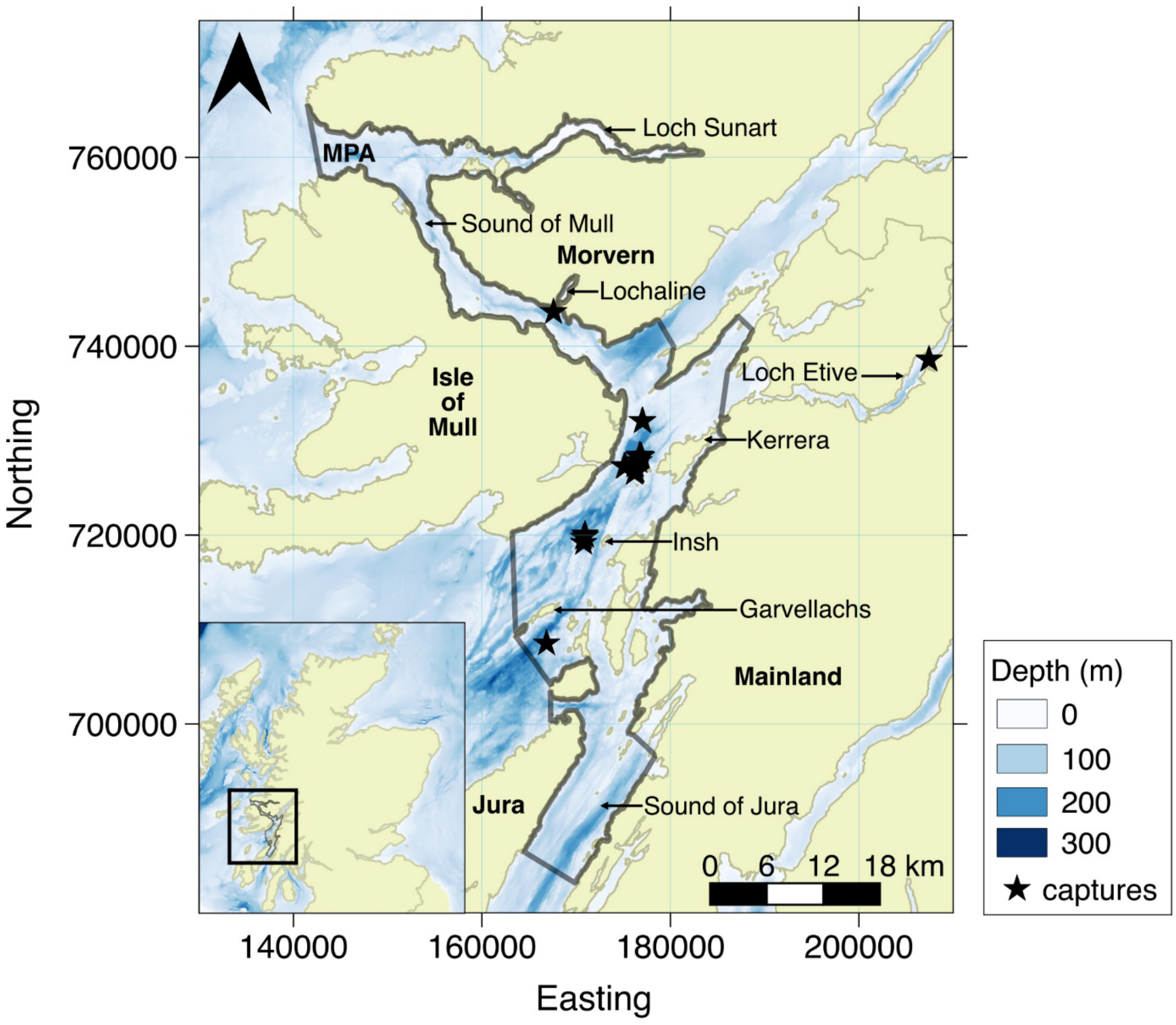

Figure S1. The study site. The inset shows the study site located within Scotland. In the main panel, the black polygon highlights the MPA. Stars mark capture sites. The bathymetry is shown across Scotland at 6 arc-second resolution and mixed 5 x 5 m and 1 arc-second resolution within the study site. Bathymetry data were sourced from Digimap and Howe *et al.* (2015). The coordinate reference system is British National Grid. Background Ordnance Survey maps © Crown copyright and database rights [2019] Ordnance Survey (100025252).

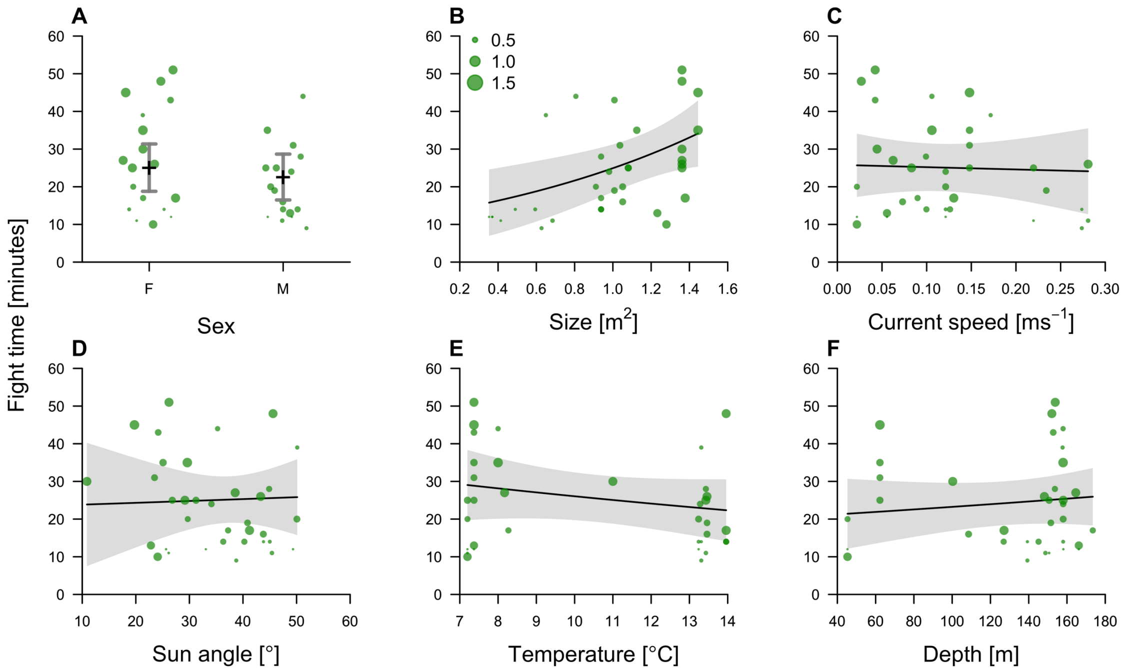

Figure S2. Fight time in relation to (A) sex, (B) body size (dorsal surface area), (C) current speed, (D) sun angle above the horizon, (E) bottom temperature and (F) the depth of the seabed. Points mark observations; point size is proportional to body size (as shown in **B**). For each variable, the black lines and surrounding intervals/envelopes mark fitted values ± 95 confidence intervals from the generalised linear model of fight time, while holding other variables constant at the first factor level or the median value. Note that confidence intervals include uncertainty in the mean as well as the effect of each explanatory variable shown. Deviance explained for this model was 32 % and standard residual diagnostics were acceptable. For coefficient estimates, see Table S5.

Supporting figures

Figures S3–S10. **Blood parameters at blood sample one (BS1) for (S3) pH, (S4) PCO_2, (S5) PO_2, (S6) bicarbonate, (S7) lactate, (S8) glucose, (S9) potassium and (S10) magnesium.** In each figure, each panel shows the relationship between the values of the blood parameter and (**A**) sex, (**B**) body size (total length), (**C**) bottom temperature, (**D**) fight time, (**E**) surface time to BS1 and (**F**) gaffing. Points mark observations. In **C** these are sized/coloured by fight time and in **D** they are sized/coloured by bottom temperature. Lines and surrounding intervals/envelopes mark predictions and 95 % confidence intervals from generalised linear models. Note that confidence intervals include uncertainty in the mean as well as the effect of each explanatory variable shown. For **C**, predictions for the effect (E) of bottom temperature (T) is shown with fight time (FT) held at its lowest (L) value (in blue) and its highest (H) value (in red), given the interaction term between these variables in the model. Likewise, for **D**, predictions for the effects of fight time are shown with bottom temperature held at its lowest value (in blue) and its highest value (in red). In other panels, predictions are shown for the following constants: sex = 'F', the median body size, the median bottom temperature, the median fight time, the median surface time and gaff = 'N'. Across all models, deviance explained ranged from 10–66 (median = 39) % (Table S8). Standard residual diagnostics were broadly acceptable.

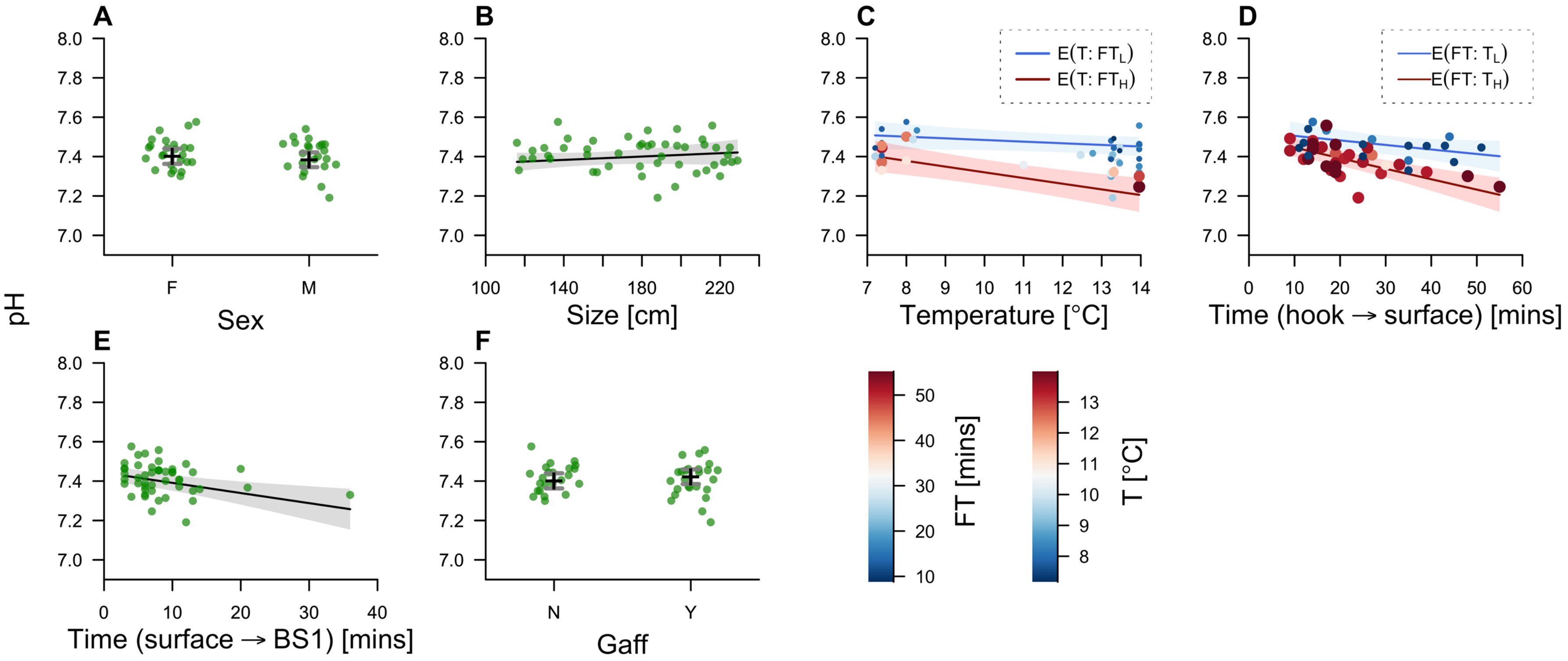

Figure S3. pH at blood sample one (BS1) in relation to (A) sex, (B) body size (total length), (C) bottom temperature, (D) fight time, (E) surface time to BS1 and (F) gaffing. Figure properties are as described in the overall caption for Figures S3–10.

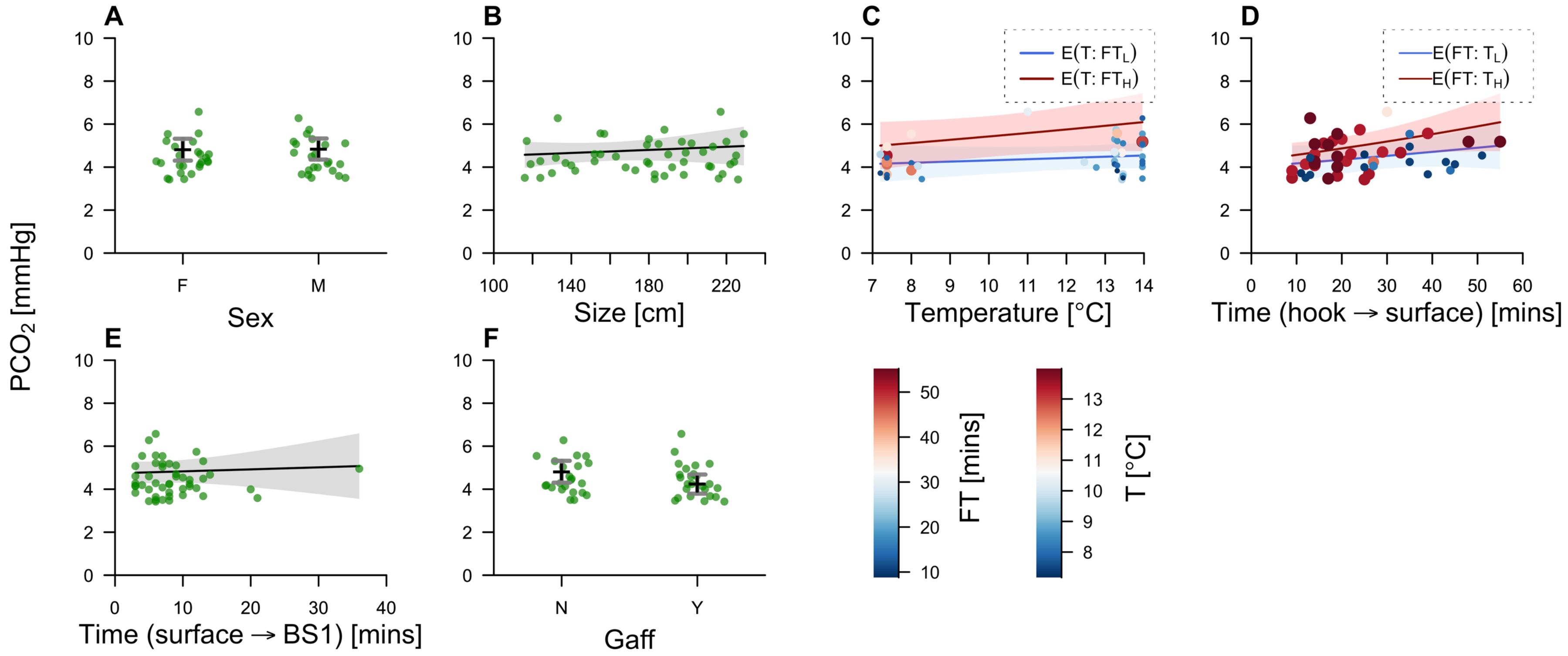

Figure S4. PCO₂ at blood sample one (BS1) in relation to (A) sex, (B) body size (total length), (C) bottom temperature, (D) fight time, (E) surface time to BS1 and (F) gaffing. Figure properties are as described in the overall caption for Figures S3–10.

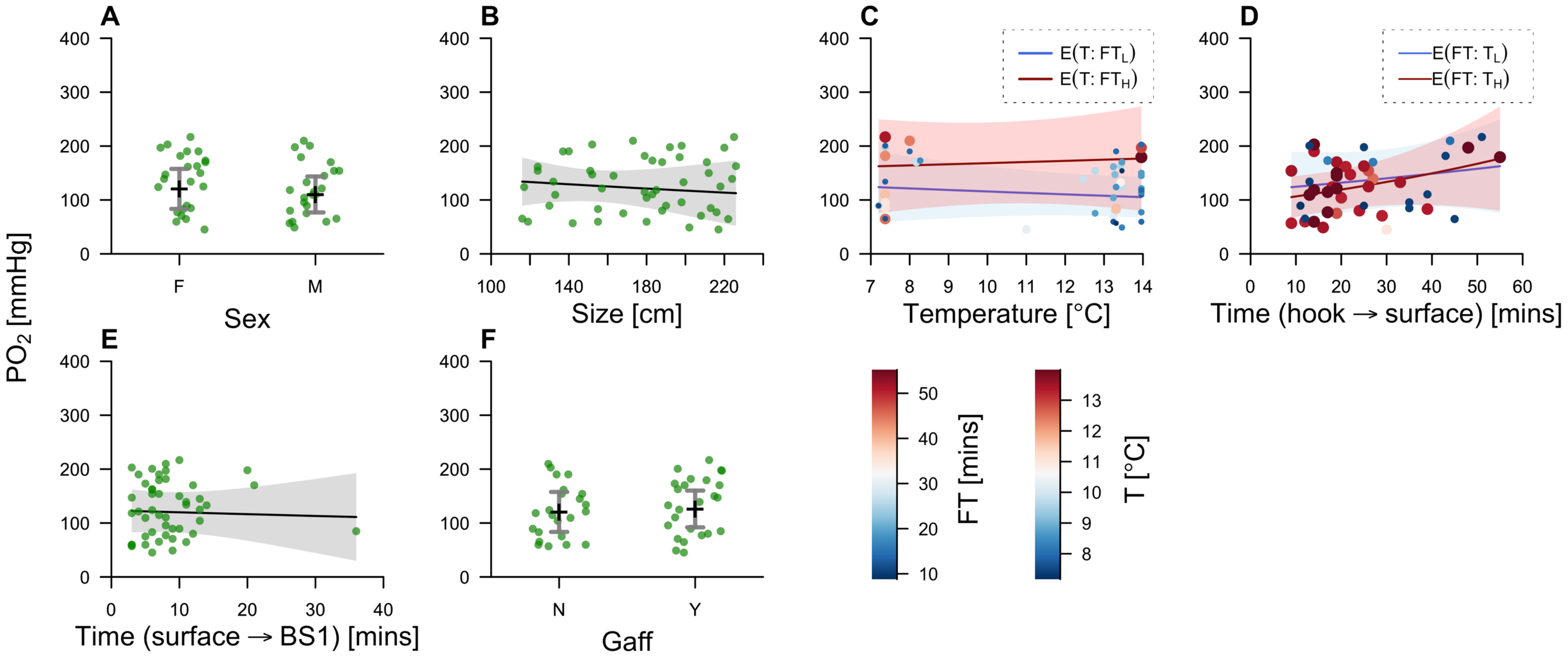

Figure S5. PO_2 at blood sample one (BS1) in relation to (A) sex, (B) body size (total length), (C) bottom temperature, (D) fight time, (E) surface time to BS1 and (F) gaffing. Figure properties are as described in the overall caption for Figures S3–10.

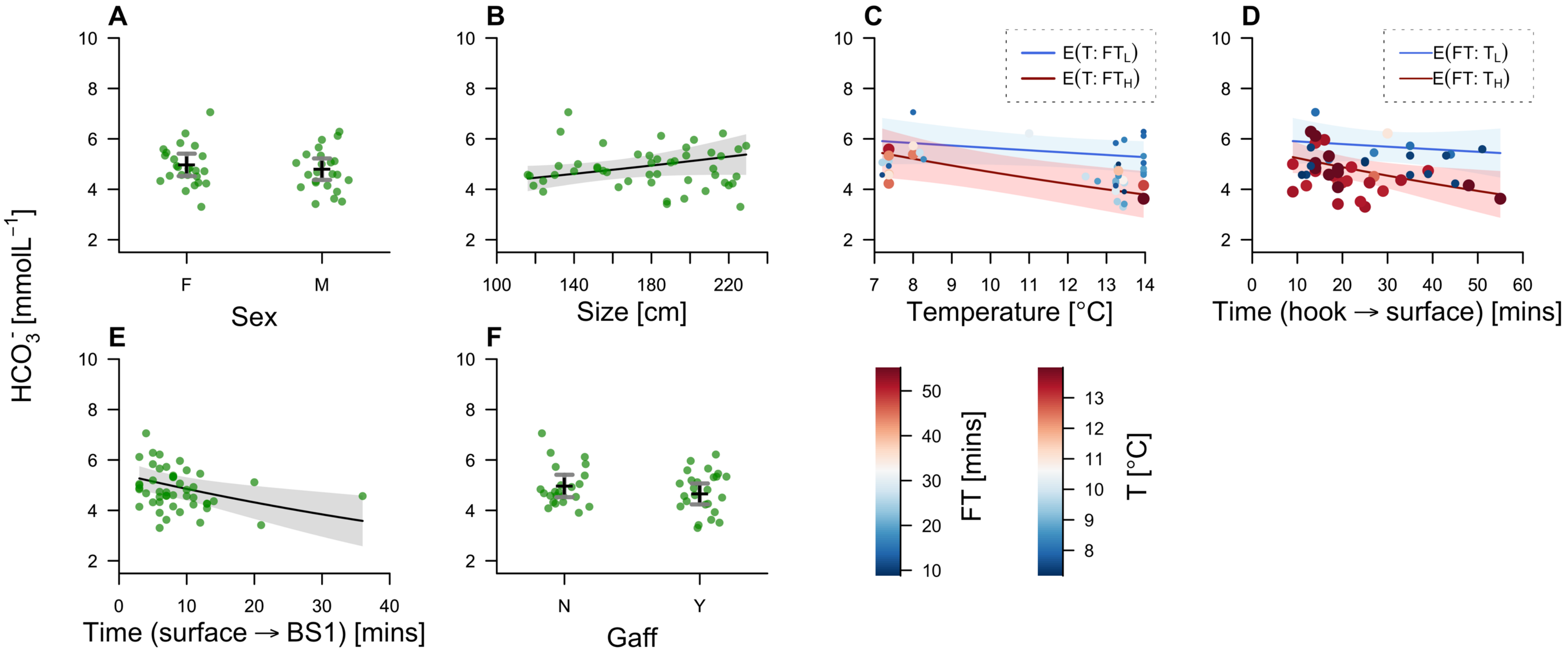

Figure S6. Bicarbonate at blood sample one (BS1) in relation to (A) sex, (B) body size (total length), (C) bottom temperature, (D) fight time, (E) surface time to BS1 and (F) gaffing. Figure properties are as described in the overall caption for Figures S3–10.

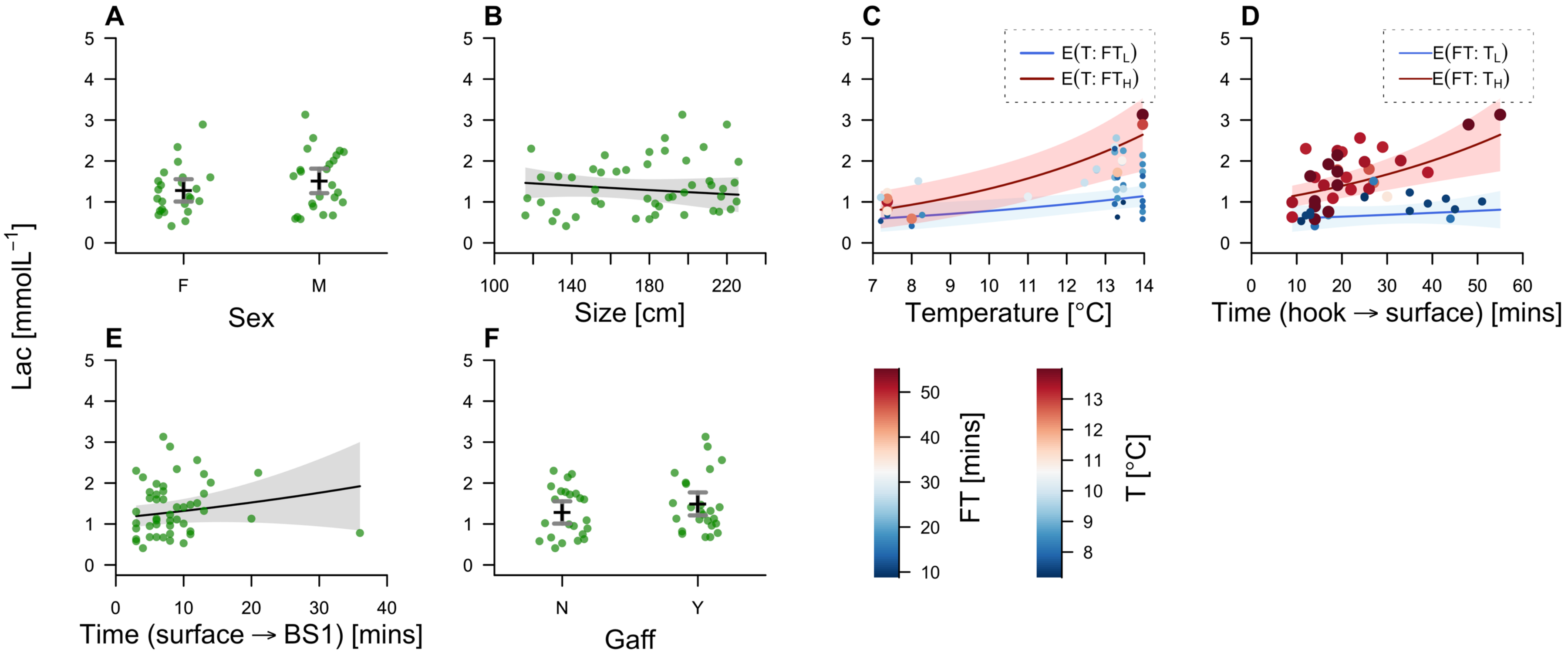

Figure S7. Lactate at blood sample one (BS1) in relation to (A) sex, (B) body size (total length), (C) bottom temperature, (D) fight time, (E) surface time to BS1 and (F) gaffing. Figure properties are as described in the overall caption for Figures S3–10.

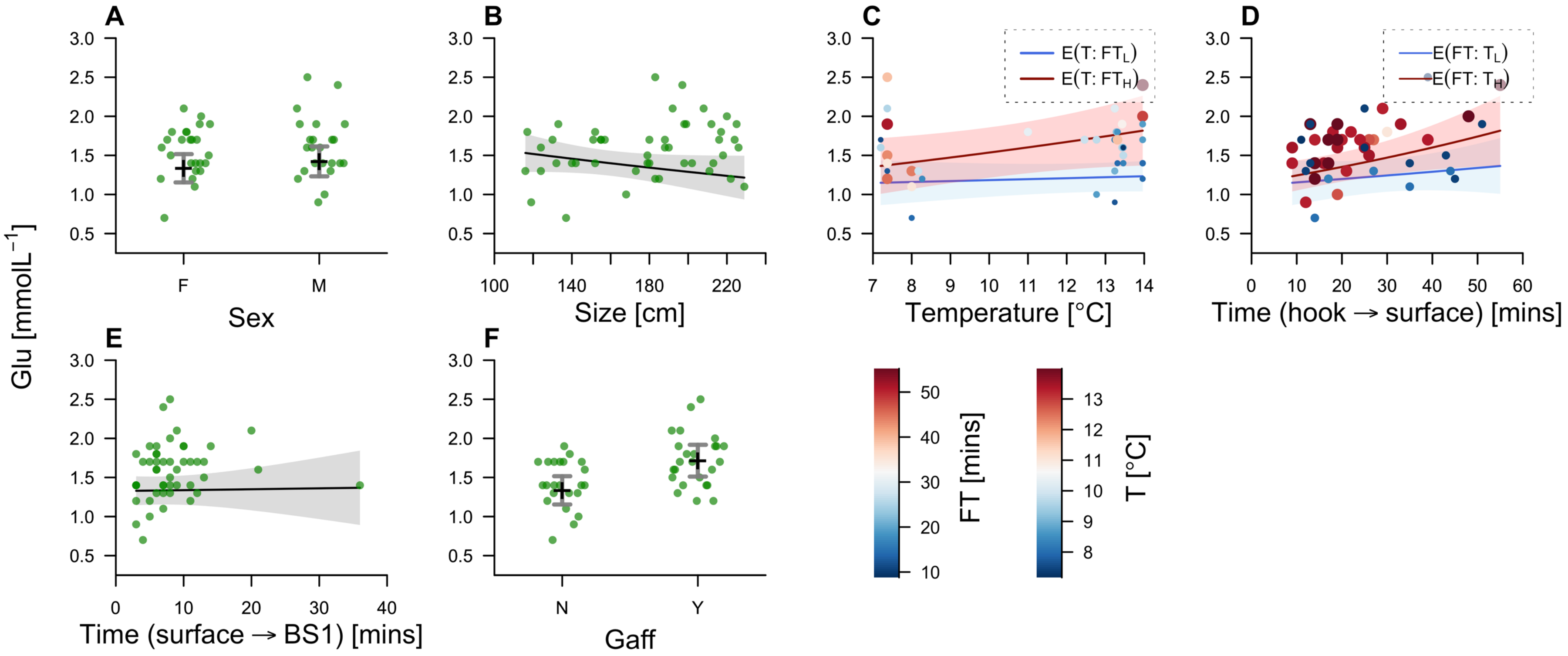

Figure S8. Glucose at blood sample one (BS1) in relation to (A) sex, (B) body size (total length), (C) bottom temperature, (D) fight time, (E) surface time to BS1 and (F) gaffing. Figure properties are as described in the overall caption for Figures S3–10.

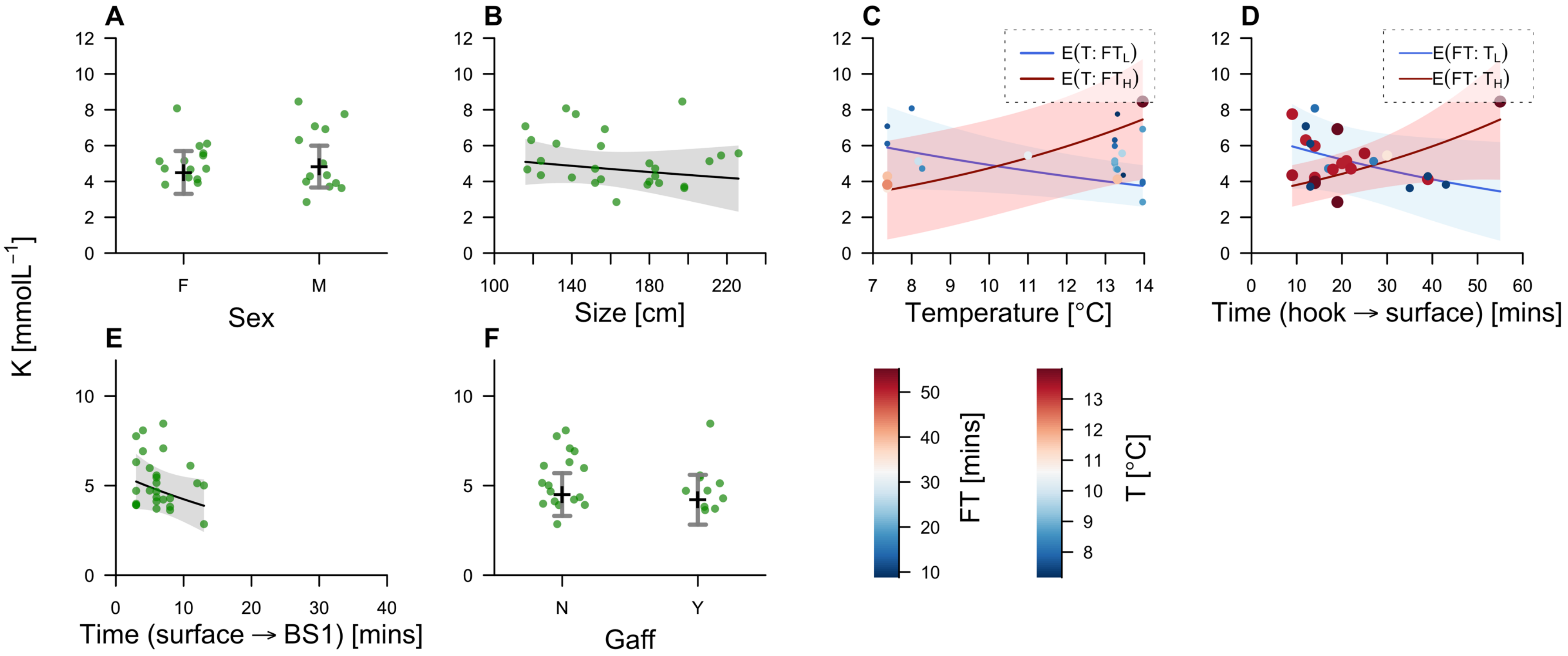

Figure S9. Potassium at blood sample one (BS1) in relation to (A) sex, (B) body size (total length), (C) bottom temperature, (D) fight time, (E) surface time to BS1 and (F) gaffing. Figure properties are as described in the overall caption for Figures S3–10.

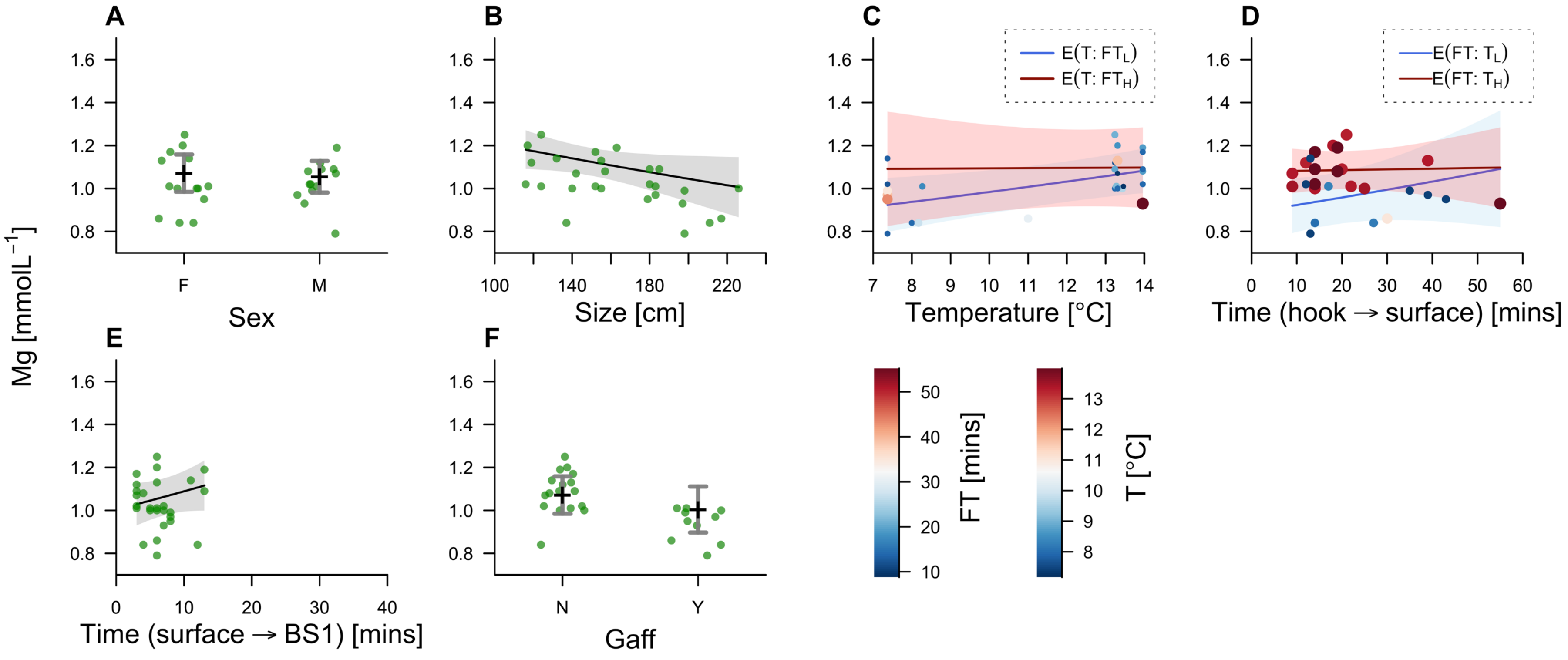

Figure S10. Magnesium at blood sample one (BS1) in relation to (A) sex, (B) body size (total length), (C) bottom temperature, (D) fight time, (E) surface time to BS1 and (F) gaffing. Figure properties are as described in the overall caption for Figures S3–10.

Figures S11–16. Blood parameters at blood sample two (BS2) for (S11) pH, (S12) PCO_2, (S13) PO_2, (S14) bicarbonate, (S15) lactate and (S16) glucose in relation to sex, body size, bottom temperature, fight time, surface time to BS2, gaffing and surgery. Figure properties follow those for Figures S3–10 and are shown for each explanatory variable with surgery held constant at surgery = 'N' and other variables held constant at the values described previously. Percentage deviance explained ranged from 27–50 (median = 34) % (Table S9). Model residual diagnostics were broadly acceptable, with no significant deviations from normality for any blood parameter (Table S3).

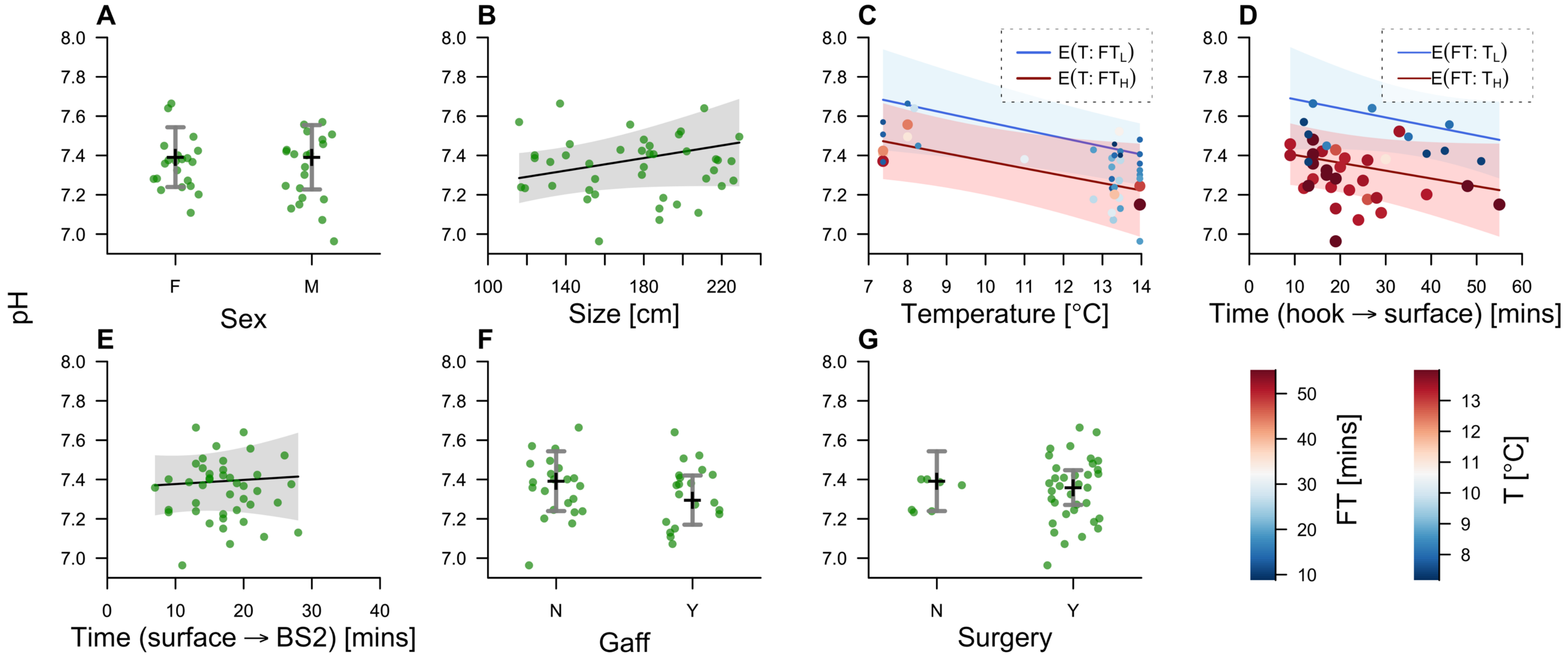

Figure S11. pH at blood sample two (BS2) in relation to (A) sex, (B) body size (total length), (C) bottom temperature, (D) fight time, (E) surface time to BS2, (F) gaffing and (G) surgery. Figure properties are as described in the overall caption for Figures S11–16.

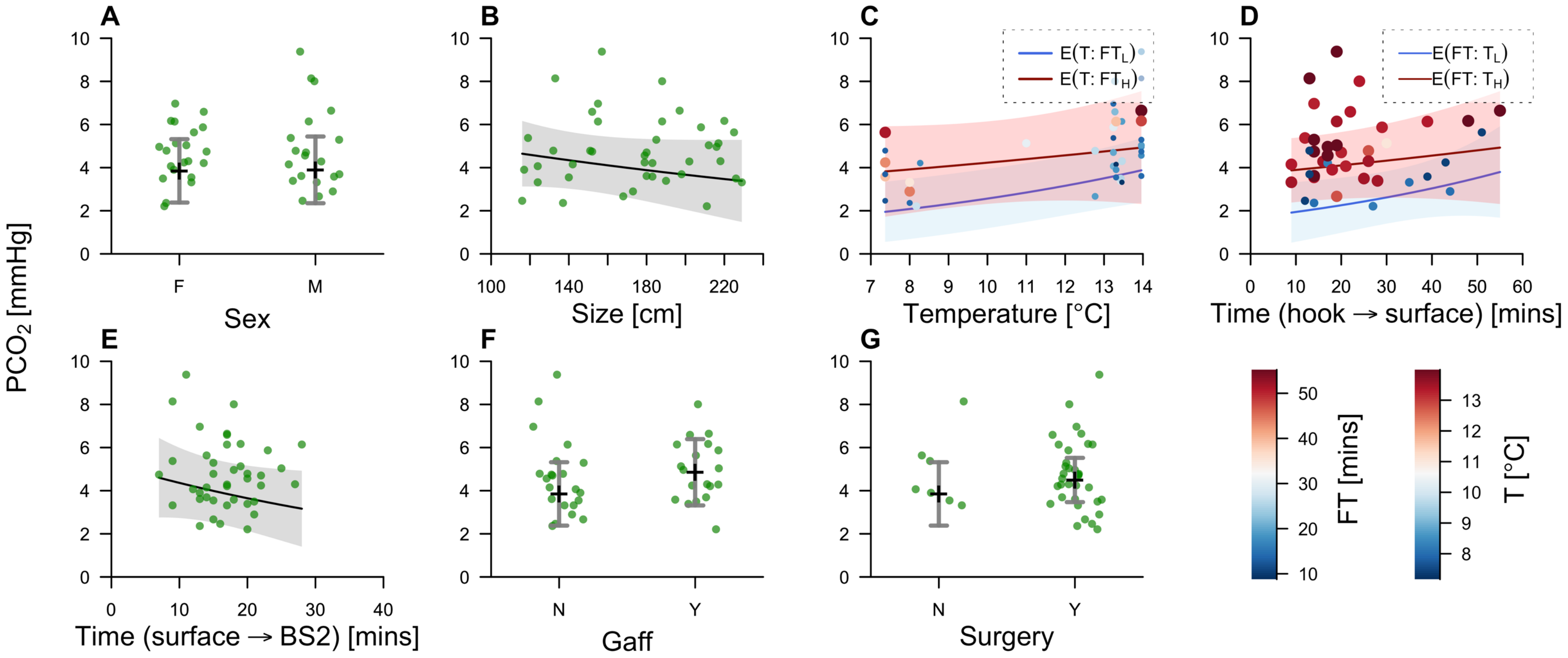

Figure S12. PCO_2 at blood sample two (BS2) in relation to (A) sex, (B) body size (total length), (C) bottom temperature, (D) fight time, (E) surface time to BS2, (F) gaffing and (G) surgery. Figure properties are as described in the overall caption for Figures S11–16.

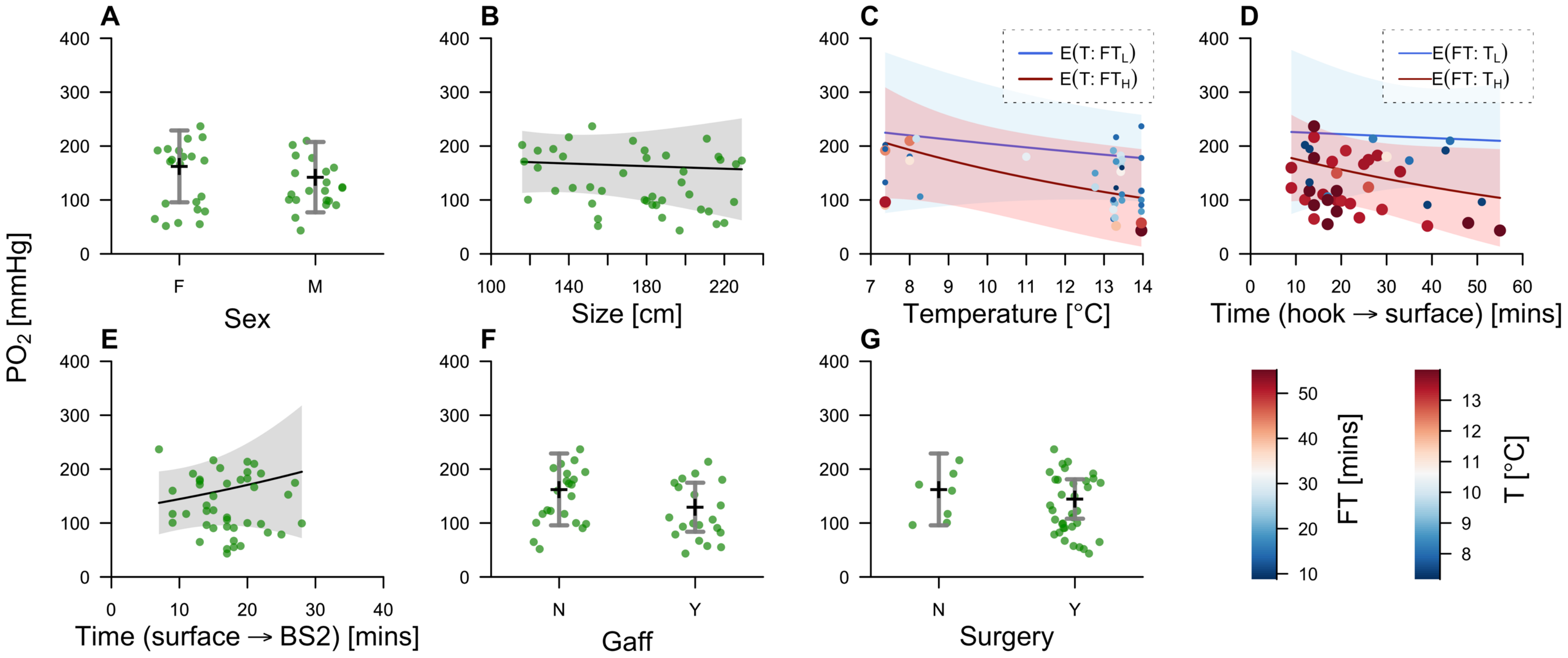

Figure S13. PO_2 at blood sample two (BS2) in relation to (A) sex, (B) body size (total length), (C) bottom temperature, (D) fight time, (E) surface time to BS2, (F) gaffing and (G) surgery. Figure properties are as described in the overall caption for Figures S11–16.

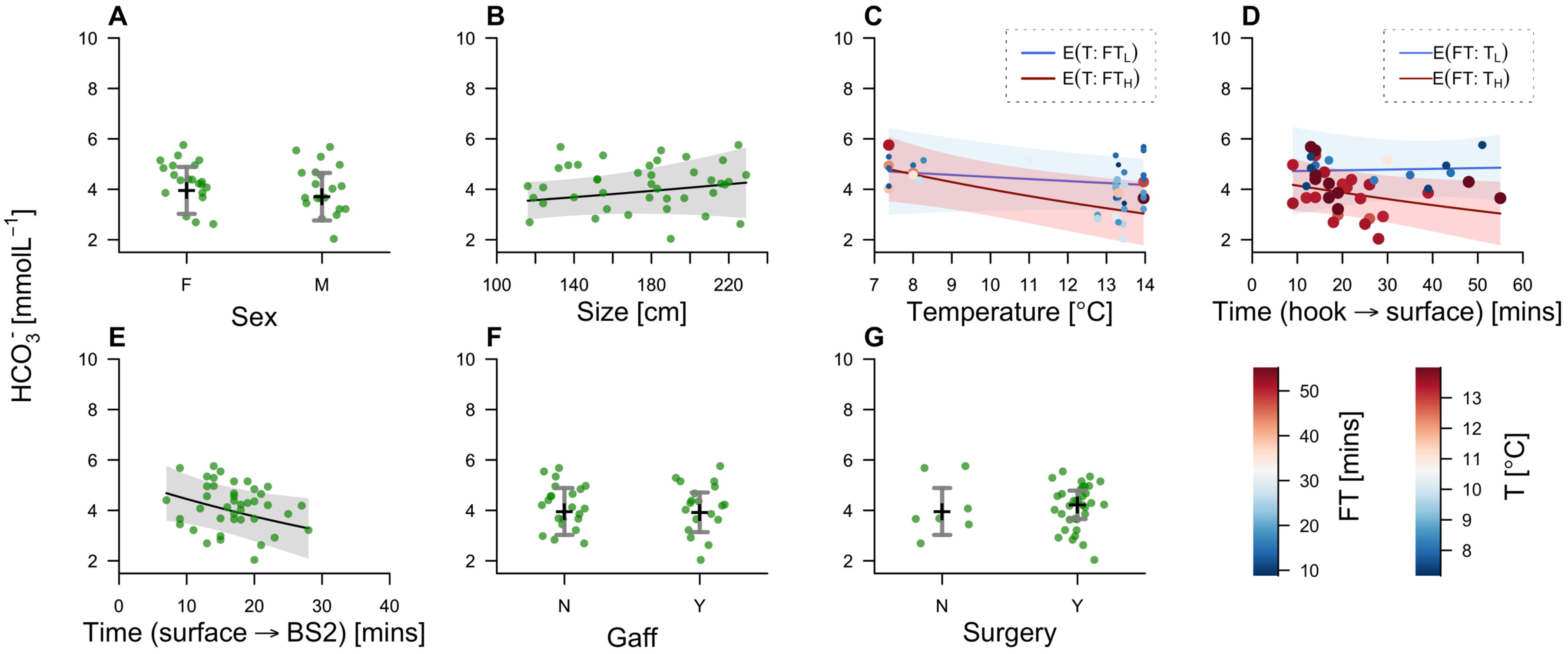

Figure S14. Bicarbonate at blood sample two (BS2) in relation to (A) sex, (B) body size (total length), (C) bottom temperature, (D) fight time, (E) surface time to BS2, (F) gaffing and (G) surgery. Figure properties are as described in the overall caption for Figures S11–16.

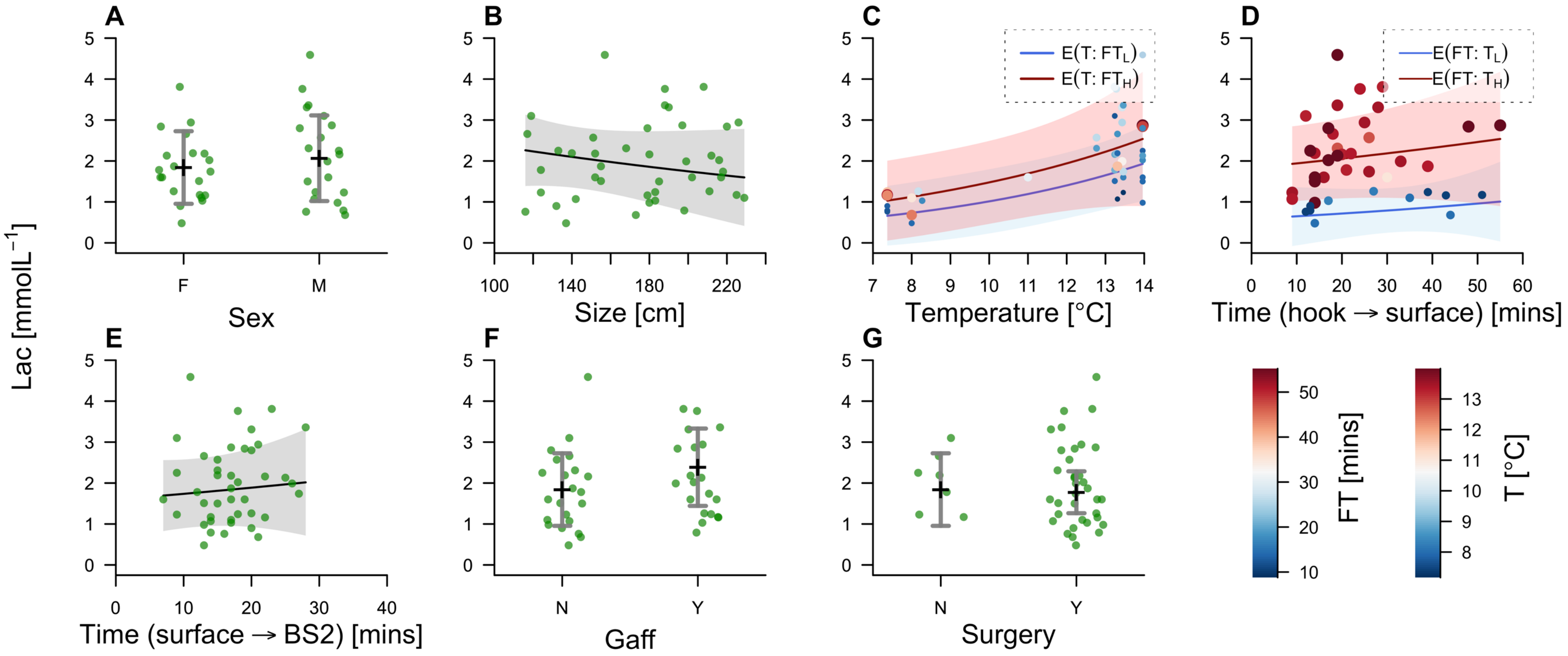

Figure S15. Lactate at blood sample two (BS2) in relation to (A) sex, (B) body size (total length), (C) bottom temperature, (D) fight time, (E) surface time to BS2, (F) gaffing and (G) surgery. Figure properties are as described in the overall caption for Figures S11–16.

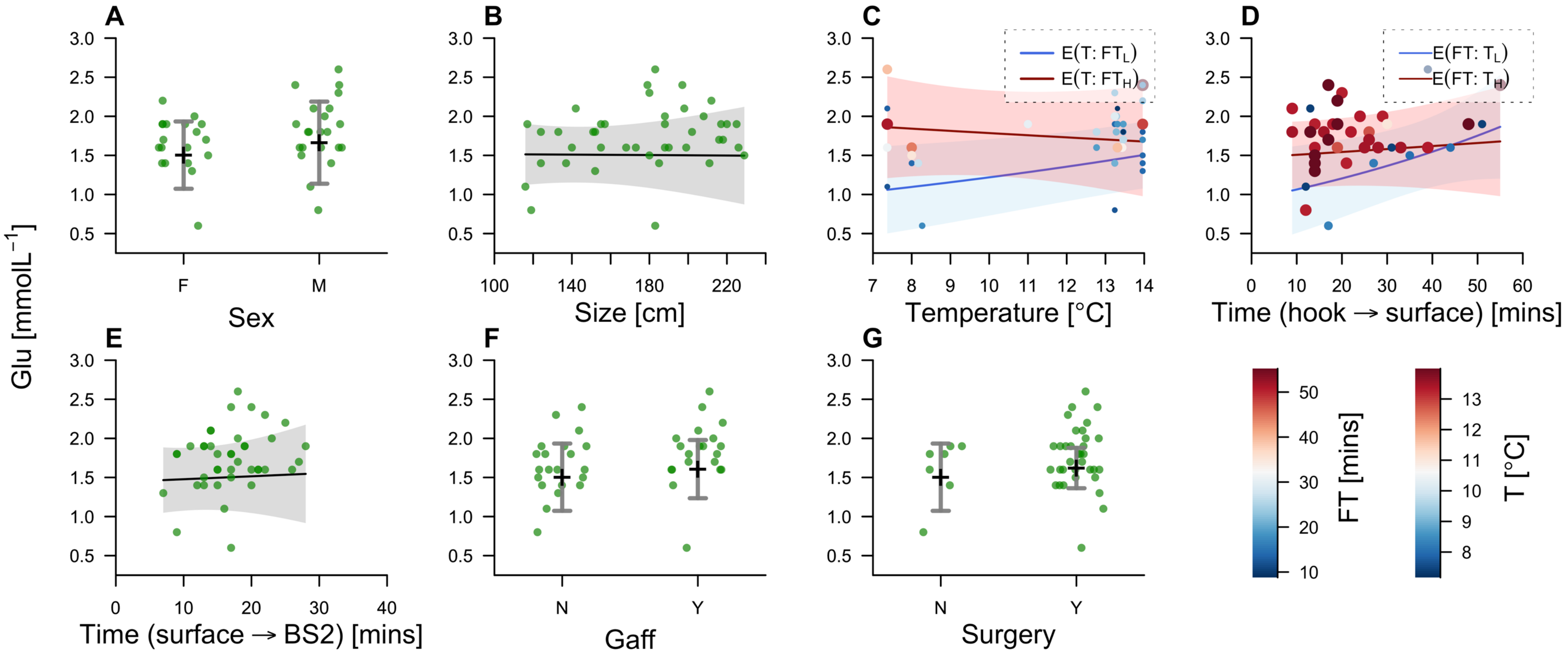

Figure S16. Glucose at blood sample two (BS2) in relation to (A) sex, (B) body size (total length), (C) bottom temperature, (D) fight time, (E) surface time to BS2, (F) gaffing and (G) surgery. Figure properties are as described in the overall caption for Figures S11–16.

Figures S17–20. The change in blood parameters from blood sample one to blood sample two (BS2 – BS1) in relation to sex, body size (total length), bottom temperature, fight time, time from the surface to BS1, time from BS1 to BS2, gaffing and surgery. Figure properties follow those for Figures S3–16. Deviance explained for these models ranged from 18–39 (median = 35) % (Table S11). Model residual diagnostics were broadly acceptable.

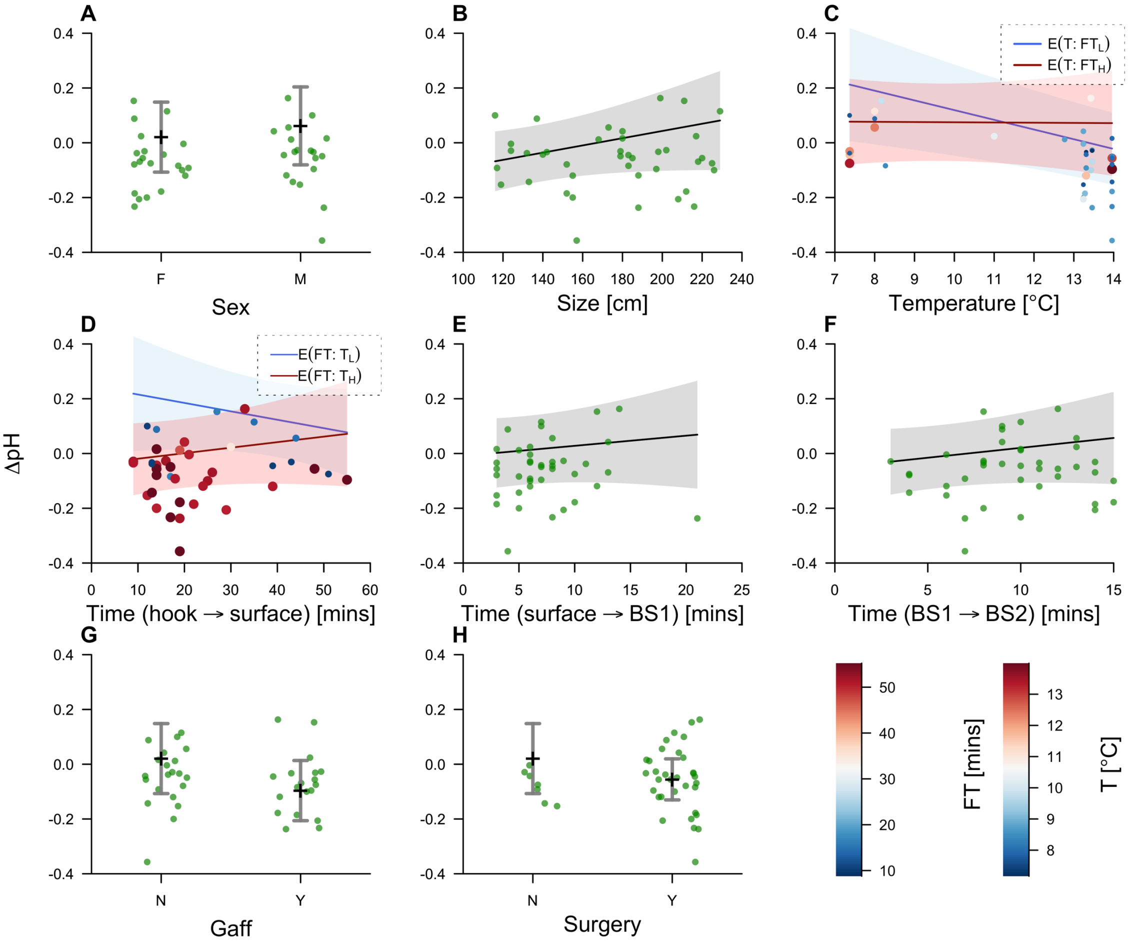

Figure S17. The change in pH from BS1 to BS2 (BS2 – BS1) in relation to (A) sex, (B) body size (total length), (C) bottom temperature, (D) fight time, (E) surface time to BS1, (F) time from BS1 to BS2, (G) gaffing and (H) surgery. Figure properties are as described in the overall caption for Figures S17–20.

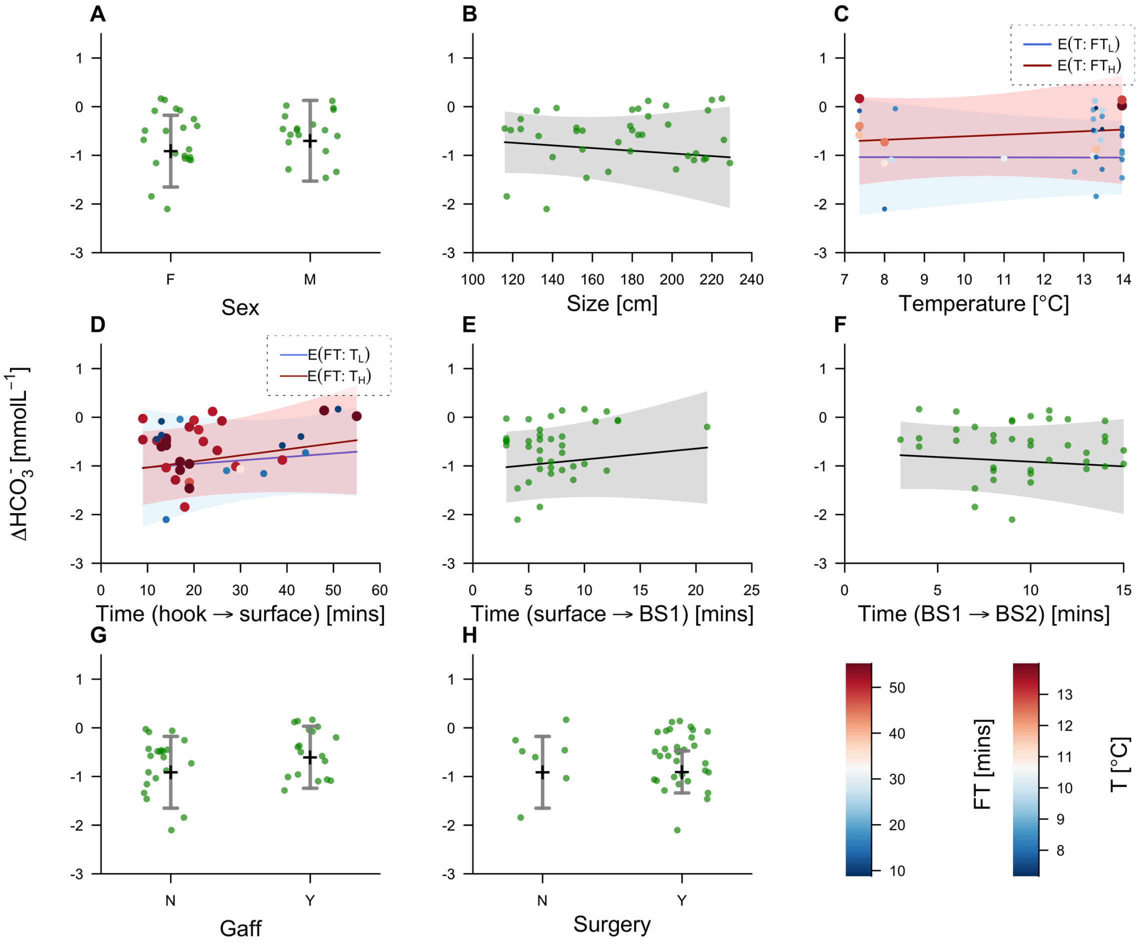

Figure S18. The change in bicarbonate from BS1 to BS2 (BS2 − BS1) in relation to (A) sex, (B) body size (total length), (C) bottom temperature, (D) fight time, (E) surface time to BS1, (F) time from BS1 to BS2, (G) gaffing and (H) surgery. Figure properties are as described in the overall caption for Figures S17–20.

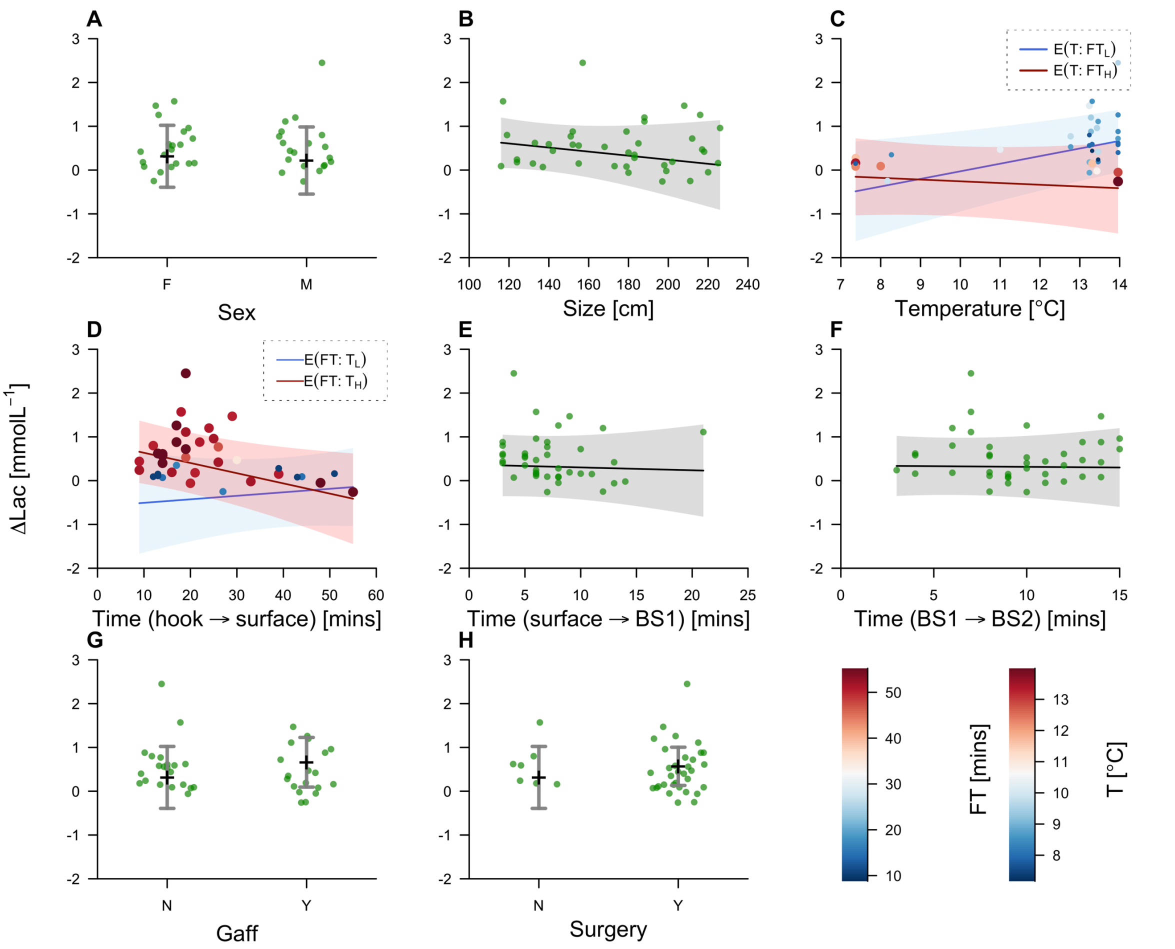

Figure S19. The change in lactate from BS1 to BS2 (BS2– BS1) in relation to (A) sex, (B) body size (total length), (C) bottom temperature, (D) fight time, (E) surface time to BS1, (F) time from BS1 to BS2, (G) gaffing and (H) surgery. Figure properties are as described in the overall caption for Figures S17–20.

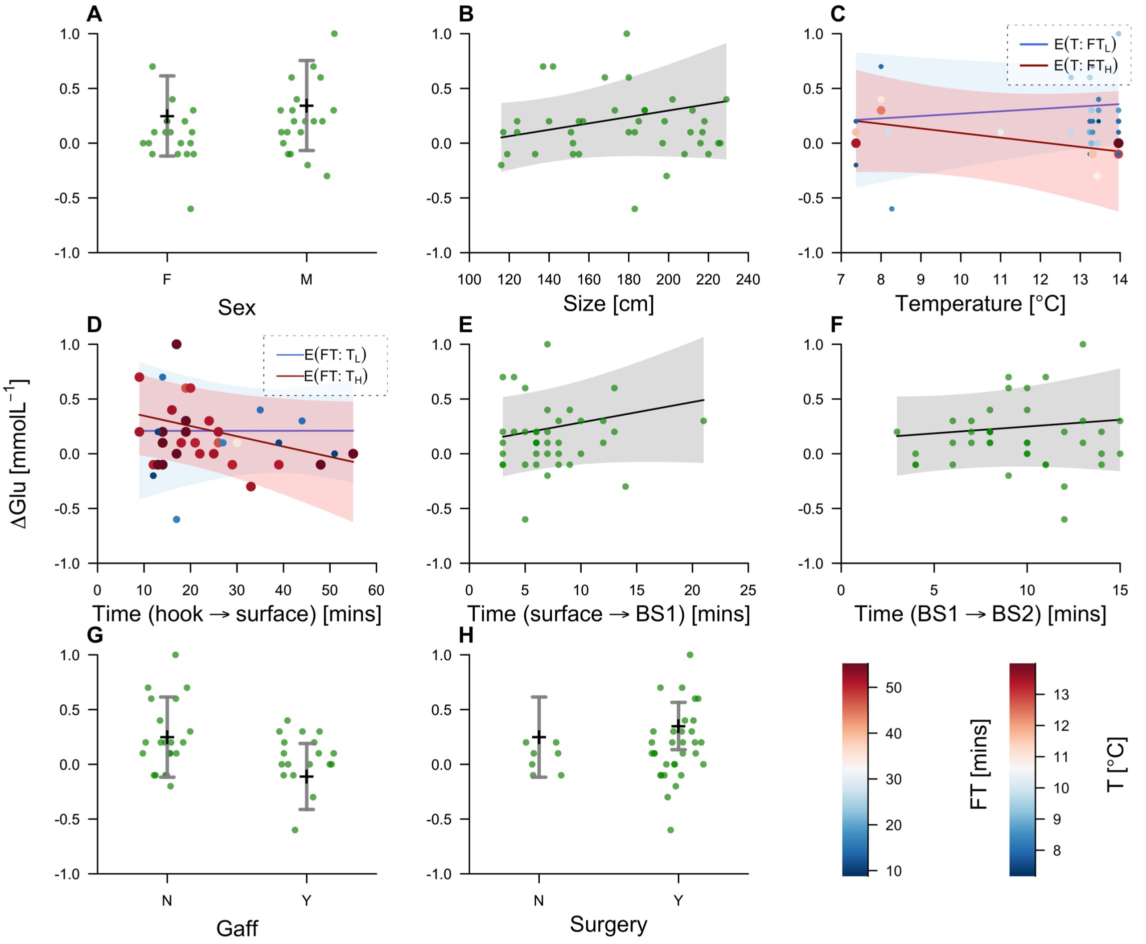

Figure S20. The change in glucose from BS1 to BS2 (BS2 − BS1) in relation to (A) sex, (B) body size (total length), (C) bottom temperature, (D) fight time, (E) surface time to BS1, (F) time from BS1 to BS2, (G) gaffing and (H) surgery. Figure properties are as described in the overall caption for Figures S17–20.

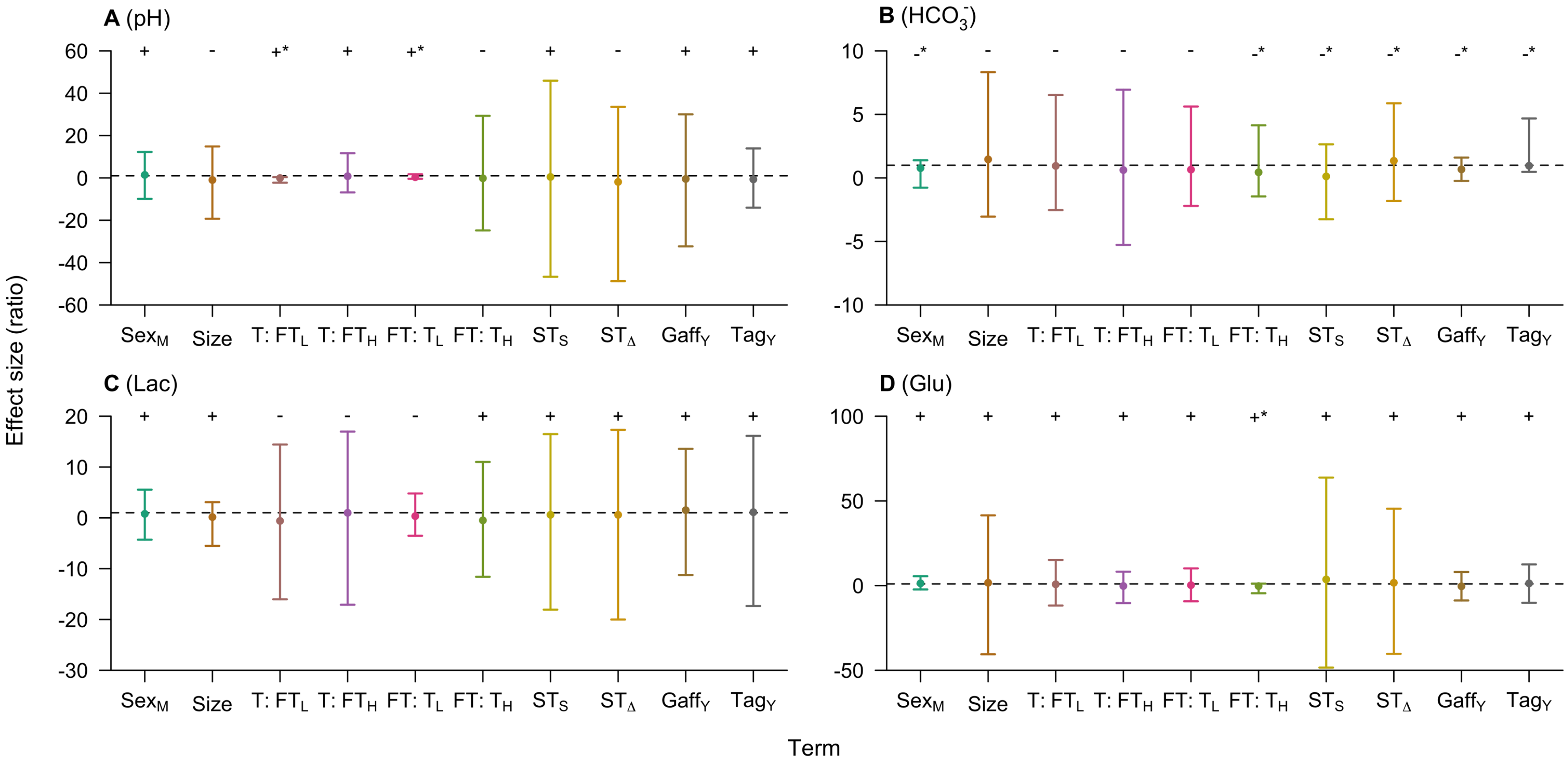

Figure S21. Effect ratios for the change in blood parameters from blood sample one to blood sample two (BS2 − BS1), following Figures 2–3. (**A**) pH, (**B**) bicarbonate, (**C**) lactate and (**D**) glucose were included in this analysis. As in Figures 2–3, in each panel, points show the mean effect size of specific explanatory variable ± 95 % confidence intervals (vertical bars) based on 5,000 bootstrap simulations. Effect ratios are defined as the ratio between simulated values of the change in the blood parameter at the second, versus the first, factor level (for sex, gaffing and

tagging), or the highest, versus the lowest, value (for continuous explanatory variables), while holding other variables constant. (For example, values for Sex_M represent the ratio between the change in blood parameter values for males versus females, with other variables held constant at the first factor level or the median value: see Supporting Information §3.5). For each blood parameter, the following effects are shown: the effect of being male (Sex_M), the effect of size (total length), the effect of temperature when fight time is low ($T{:}\,FT_L$), the effect of temperature when fight time is high ($T{:}\,FT_H$), the effect of fight time when temperature is low ($FT{:}\,T_L$), the effect of fight time when temperature is high ($FT{:}\,T_H$), the effect of surface time to BS1 (ST_S), the effect of surface time from BS1 to BS2 (ST_Δ), the effect of gaffing ($Gaff_Y$) and the effect of tagging (Tag_Y). The + and – signs above each bar indicate whether the baseline (or 'starting') prediction (e.g., the average predicted change for *females* or the change for the *smallest* individuals) was significantly (*) positive (+) or negative (-). This facilitates interpretation of each effect size, since effect sizes for the *change* in blood parameters (unlike in Figures 2–3) can be positive or negative: ratio = 1 implies no change, ratio > 1 implies an absolute increase; ratio < 1 implies an absolute decrease; and ratio < 0 implies a switch in the direction of change. For example, for pH, the average predicted baseline change is marginally positive for $T{:}\,FT_L$ (estimate = 0.22 [0.02–0.53]), see Figure S17); i.e., at the coldest temperatures (7.20 °C) and the lowest fight times (8 minutes), and when other variables are held constant at the first factor level or median value, on average pH is expected to increase fractionally from BS1 to BS2. However, at the warmest temperatures (13.96 °C), the distribution of ratios (ratio = -0.072 [-2.26–0.39]) suggests smaller increases or (more likely) decreases in pH from BS1 to BS2 (in line with the predicted change in pH with increasing temperature at low fight times shown in Figure S17).

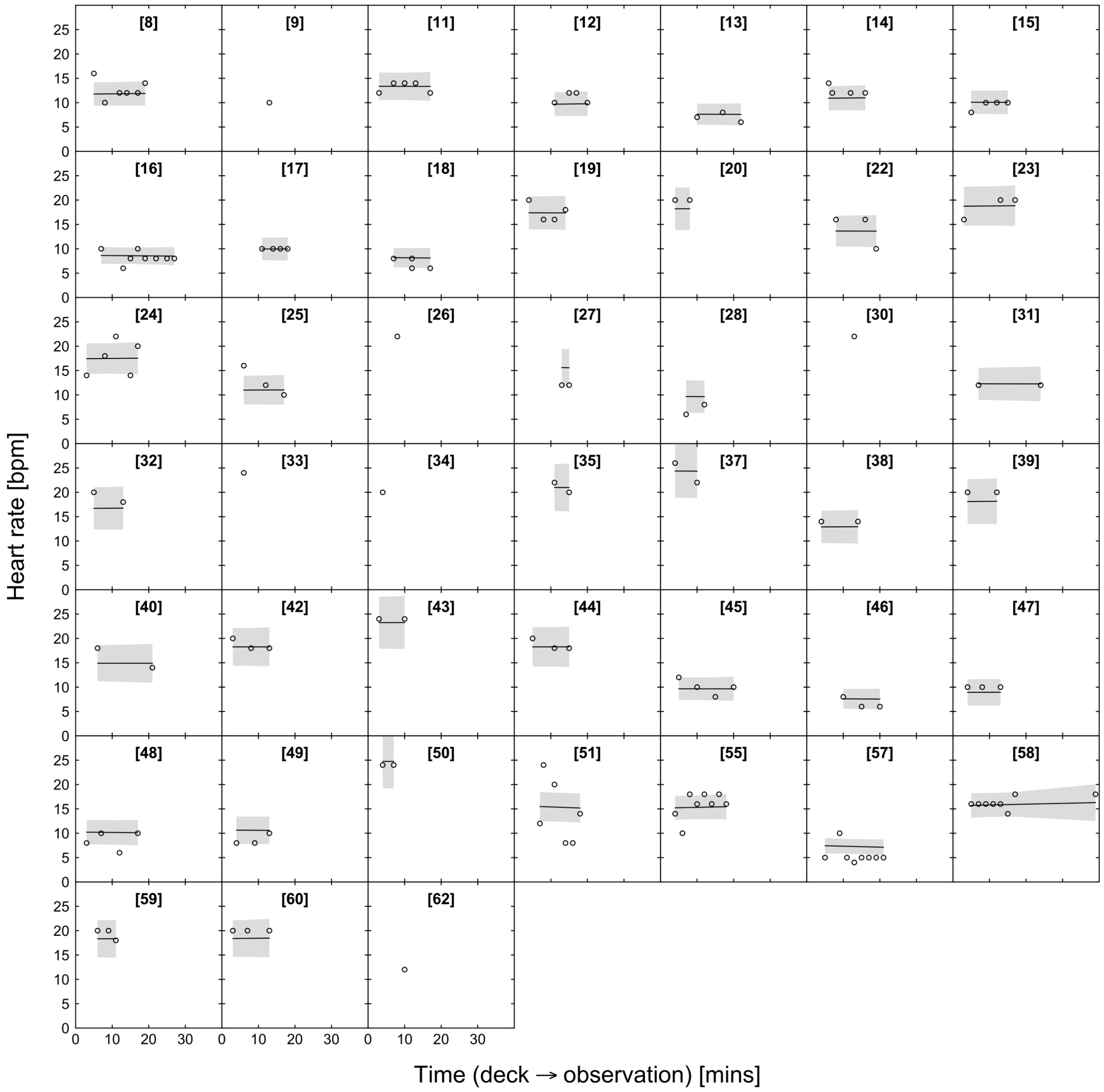

Figure S22. Trends in heart rates during time on deck. Each panel shows the observed heart rates (points) during a specific handling event (with panel titles following Table S1). The black line and surrounding envelope show predictions and 95 % pointwise confidence bands from the negative binomial generalised additive random effects model of heart rates. Note that observations were recorded irregularly through time and the number of observations differs among events. For coefficient estimates, see Table S12.

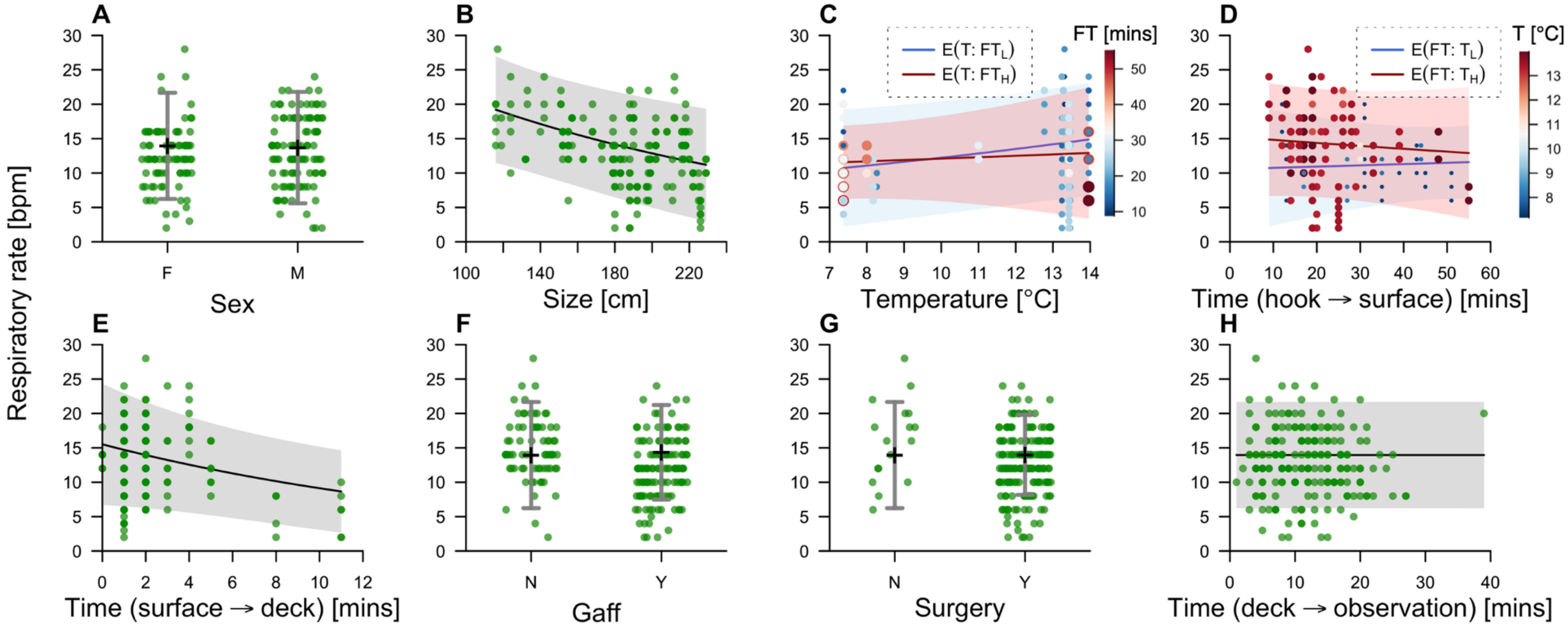

Figure S23. Respiratory rate in relation to (A) sex, (B) body size (total length), (C) bottom temperature, (D) fight time, (E) surface time, (F) gaffing, (G) surgery and (H) deck time. Following Figure 4, points mark observations for each event. In **C** and **D** these are sized/coloured by fight time and temperature respectively. Black lines and surrounding bars/envelopes mark predictions and 95 % confidence intervals across the range of values of each explanatory variable, with other variables held constant at the first factor level or median value, apart from in **C** and **D** in which the predicted effects of bottom temperature (T) and fight time (FT) are shown at both the lowest (L) and highest (H) value of the other variable, given the interaction term between these variables in the model. Note that confidence intervals include uncertainty in the effect of the explanatory variable and the mean. For both heart rates (Figure 4) and respiratory rates (this figure), residual diagnostic checks showed notable evidence of non-normality, but individuals' responses during handling on deck were accurately predicted (Figures S22 and S24). For coefficient estimates, see Table S13.

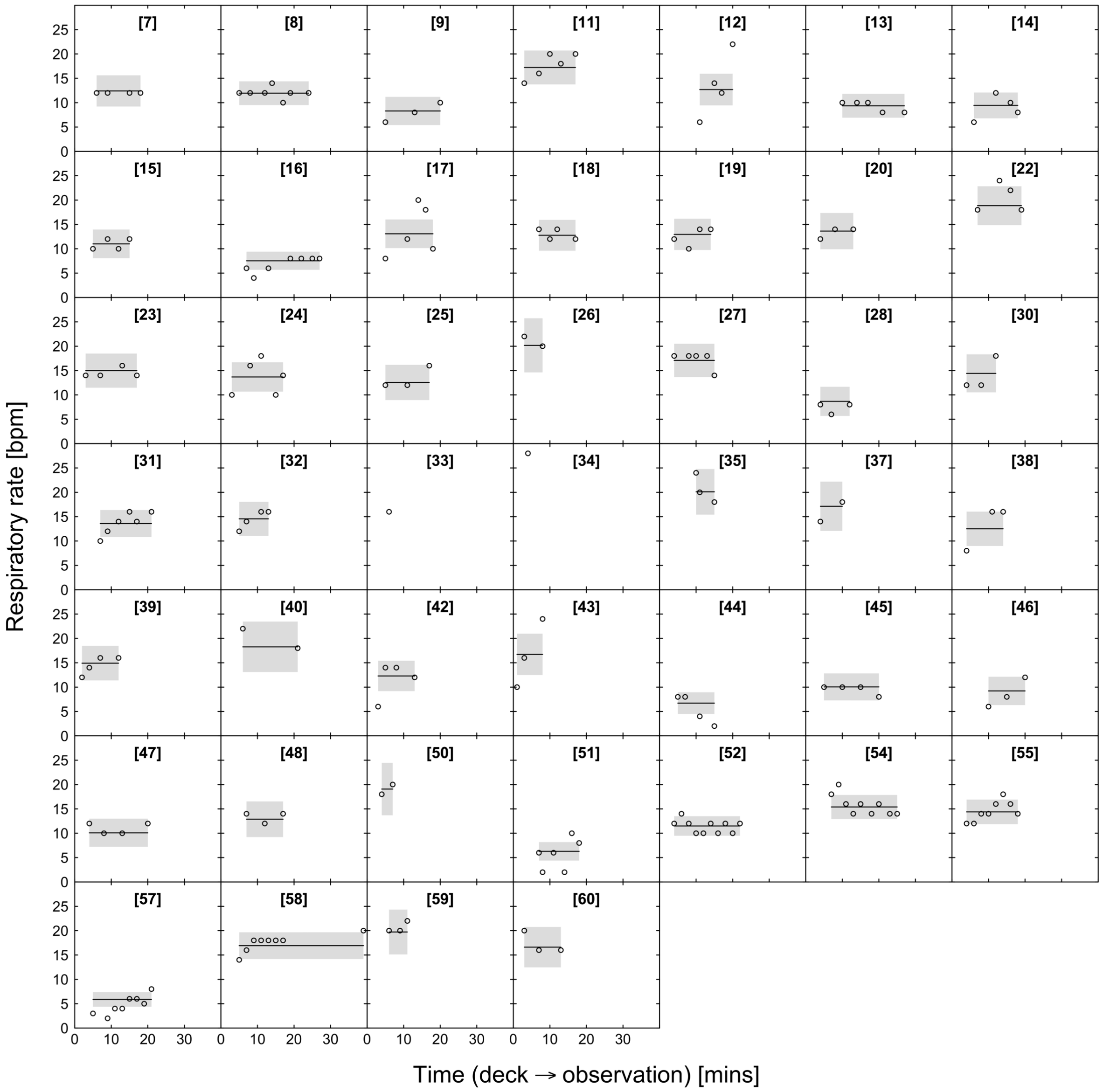

Figure S24. Trends in respiratory rates during time on deck. Each panel shows the observed respiratory rates (points) during a specific handling event (with panel titles following Table S1). The black line and surrounding envelope show predictions and 95 % pointwise confidence bands from the negative binomial generalised additive random effects model of respiratory rates. Note that observations were recorded irregularly through time and the number of observations differs among events. For coefficient estimates, see Table S13.

Supporting tables

Table S1. Summary of capture events. For each event (ID), the date (year-month-day), the coordinates on the British National Grid, the captured individual's passive integrated transponder identifier, sex, total length (cm), disc width (cm) and whether (1) or not (0) the individual was acoustically tagged is shown. Note that two events[a] represent the same individual. Sorted by date.

ID	Date	Easting	Northing	PIT	Sex	TL	DW	Tag
1	20-03-20	176656.9	727852.3	31199293	F	130	91	0
2	20-03-20	176656.9	727852.3	31199309	M	191	147	0
3	20-03-19	207446.9	738604.2	31199256	F	213	160	0
4	20-03-19	207446.9	738604.2	31199492	F	112	84	0
5	20-03-19	207446.9	738604.2	1972215	F	173	135	0
6	20-03-06	176931.6	728330.4	7093185	F	213	165	0
7	20-03-06	176865.6	728369.2	7093370	F	132	98	1
8	20-03-06	176865.6	728369.2	2054522	F	179	142	1
9[a]	20-03-06	176831.7	728352.4	29241467	F	225	165	0
10	20-03-05	176217.7	727559.4	07092889	M	163	127	0
11	20-03-05	176750.0	728425.5	29241562	M	116	86	1
12	20-03-05	176750.0	728425.5	00372268	M	198	157	1
13	20-03-04	177044.3	732094.2	2924	F	222	170	1
14	20-03-04	177044.3	732094.2	03700289	M	198	150	1
15	20-03-04	177044.3	732094.2	29241470	M	183	137	1
16	20-03-04	177044.3	732094.2	29241480	M	192	147	1
17	20-03-04	177044.3	732094.2	407092918	M	188	144	1
18	19-11-18	156195.5	653861.3	29241334	F	217	165	1
19	19-08-14	170792.2	719736.6	29241545	M	180	137	1
20	19-08-14	170801.9	719189.5	02070420	M	185	137	1
21	19-08-14	170562.3	715473.3	29241491	F	118	84	0
22	19-08-13	-	-	29241473	F	212	157	1
23	19-08-13	-	-	29241363	F	152	112	1
24	19-08-13	-	-	29241510	M	179	128	1
25	19-08-13	176733.2	728262.8	07093367	F	220	165	1
26	19-08-12	-	-	29241483	M	133	91	0
27	19-08-12	-	-	07093357	M	157	117	1
28	19-08-12	-	-	070932939	M	197	152	1
29	19-08-12	-	-	07092986	M	193	151	0
30	19-08-12	-	-	07093226	M	163	122	0

Supporting tables

ID	Date	Easting	Northing	PIT	Sex	TL	DW	Tag
31	19-08-08	167606.1	743639.9	07092980	F	216	166	1
32	19-08-07	176281.8	726741.9	02052433	F	155	114	1
33	19-08-07	176201.4	726605.0	07092920	F	140	99	0
34	19-08-07	176201.4	726605.0	07093095	F	117	81	0
35	19-08-07	176201.4	726605.0	02020995	M	142	112	1
36	19-08-07	176201.4	726605.0	00372316	F	203	155	0
37	19-09-06	176326.7	726849.2	29241556	M	119	86	0
38	19-08-06	176315.2	726847.9	02103977	F	208	155	1
39	19-08-06	176315.2	726847.9	29241524	F	152	114	1
40	19-08-06	176315.2	726847.9	10988867	M	188	140	1
41	19-08-06	176315.2	726847.9	07093005	M	180	135	0
42	19-08-06	176315.2	726847.9	02022877	F	155	109	1
43	19-08-06	176314.8	726838.7	07092910	F	124	89	0
44	19-08-06	176315.2	726847.9	07093118	M	180	145	1
45	19-04-04	170945.8	720094.5	7093330	F	183	137	1
46	19-04-04	170997.1	719915.2	29241486	F	211	165	1
47[a]	19-04-02	176268.5	727447.1	29241467	F	229	170	1
48	19-04-02	176268.5	727447.1	00366757	M	173	127	1
49	19-04-02	176268.5	727447.1	02143576	F	137	102	1
50	18-08-22	-	-	7092885	M	124	91	0
51	18-08-22	175199.9	727325.2	7092921	M	188	142	1
52	18-08-22	-	-	10991061	M	199	152	1
53	18-08-22	175182.1	727261.0	7093359	M	163	117	1
54	18-08-22	175119.5	727270.1	7092902	F	218	165	1
55	18-08-21	166859.6	708548.6	3194	M	202	145	1
56	18-08-21	176239.4	727482.1	7092935	F	229	180	0
57	18-08-21	176246.5	727440.8	7093338	F	226	165	1
58	18-08-20	176799.0	728318.8	7093231	M	190	137	1
59	18-08-02	-	-	7093028	M	151	107	1
60	18-08-02	-	-	7092883	M	168	126	1
61	18-08-02	-	-	2069335	M	-	-	1
62	18-08-01	-	-	7092972	F	224	170	0

Supporting tables

Table S2. Equipment, consumables and drugs. For each item listed in the methods section of the main manuscript the product details are listed.

**Item**	**Model/make or drug concentration**	**Manufacturer**	**Address**
Lidocaine hydrochloride	2% w/v solution for injection	Accord Healthcare Limited	North Harrow, Middlesex, UK
Acoustic tag	V16	Innovasea	Bedford, Nova Scotia, Canada
Acoustic tag	V13	Innovasea	Bedford, Nova Scotia, Canada
Monofilament absorbable suture	MonocrylTM, poliglecaprone 25	Ethicon	Raritan, New Jersey, United States
Hand-held blood analyser	i-STAT	Abaxis Veterinary Diagnostics	Union City, California 94587, USA
Glucometer	Accu-Check®	Roche Diagnostics	Risch-Rotkreuz, Switzerland
Bench-top biochemistry analyser	AU480 Chemistry analyzer	Beckman Coulter Diagnostics	Brea, California
Ultrasound	Easi-Scan	IMV imaging	Bellshill, Scotland, ML4 3NJ

Table S3. A summary of blood parameter values for blood sample (1) and (2) and the median heart and respiratory rate for each capture event. Blood parameter variable names are as follows: pH, carbon dioxide partial pressure (PCO_2), oxygen partial pressure (PO_2), bicarbonate (HCO_3^-), lactate (Lac), glucose (Glu), potassium (K) and magnesium (Mg). Units for PCO_2 and PO_2 are mmHg; units for HCO_3^-, lactate, glucose, K and Mg are mmolL^{-1}; and units for heart rates and respiratory rates are beats per minute and breaths per minute respectively. For one event[a], a highly implausible pH value for BS1 (7.99) was recorded; this value excluded from analysis. For another event[b], the value recorded for PCO_2 (1.00) was below the reading range of the i-STAT (< 5 mmHg); this value is excluded from analysis. While this is a potential source of bias, the influence of this one individual on analyses is likely to be minimal. For one additional event[c], mild haemolysis was documented; this event was retained in analyses. Some blood measurements shown in this table were taken from 'unhealthy' individuals but these are excluded from all analyses (see also Table S1). In total, for each blood parameter, we successfully obtained and analysed 28–50 measurements at BS1 and 18–43 at BS2 from healthy individuals.

ID	pH (1)	PCO_2 (1)	PO_2 (1)	HCO_3^- (1)	Lac (1)	Glu (1)	K (1)	Mg (1)	pH (2)	PCO_2 (2)	PO_2 (2)	HCO_3^- (2)	Lac (2)	Glu (2)	K (2)	Mg (2)	HR	RR
1	7.44	3.72	89.33	4.57	0.53	1.70	-	-	-	-	-	-	-	-	-	-	-	-
2	7.40	4.59	89.33	5.07	1.11	1.60	-	-	-	-	-	-	-	-	-	-	-	-
3	-	-	-	-	-	-	-	-	-	-	-	-	-	-	-	-	-	-
4	-	-	-	-	-	-	-	-	-	-	-	-	-	-	-	-	-	-
5	-	-	-	-	-	-	-	-	-	-	-	-	-	-	-	-	-	-
6	7.33	4.95	84.82	4.57	0.78	1.40	-	-	-	-	-	-	-	-	-	-	-	-
7	7.40	4.43	133.97	4.93	0.75	1.40	6.11	1.14	7.37	4.79	194.56	4.84	0.90	-	5.32	1.23	-	12
8	7.45	4.24	181.76	5.34	1.08	1.50	3.82	0.95	7.42	4.24	191.86	4.94	1.16	-	4.45	1.04	12	12
9	7.45	4.54	216.77	5.59	1.01	1.90	-	-	7.37	5.64	96.27	5.76	1.17	1.90	-	-	10	8
10	7.55	3.53	146.76	5.65	0.75	1.20	5.08	1.04	7.38	3.75	101.65	3.92	1.61	1.90	5.27	1.19	9	12
11	7.47	3.50	65.30	4.58	0.67	1.30	7.08	1.02	7.57	2.46	201.96	4.13	0.76	1.10	4.56	0.97	14	18

Supporting tables

ID	pH (1)	PCO_2 (1)	PO_2 (1)	HCO_3^- (1)	Lac (1)	Glu (1)	K (1)	Mg (1)	pH (2)	PCO_2 (2)	PO_2 (2)	HCO_3^- (2)	Lac (2)	Glu (2)	K (2)	Mg (2)	HR	RR
12	7.54	3.64	200.61	5.66	0.68	1.90	3.71	0.79	7.51	3.69	132.62	5.29	0.79	2.10	-	-	11	13
13	7.37	4.13	64.63	4.23	0.82	1.20	-	-	-	-	-	-	-	-	-	-	7	10
14	7.45	4.24	95.59	5.34	1.23	1.40	3.63	0.99	-	-	-	-	-	-	-	-	12	8
15	7.45	3.67	110.41	4.60	0.96	2.50	4.29	0.97	7.41	3.58	90.88	4.02	1.24	2.60	-	-	10	11
16	7.46	3.99	197.92	5.12	1.13	2.10	-	-	-	-	-	-	-	-	-	-	8	8
17	-	-	-	-	-	-	-	-	-	-	-	-	-	1.60	-	-	10	12
18	7.36	6.57	45.23	6.22	1.13	1.80	5.45	0.86	7.38	5.13	180.19	5.15	1.60	1.90	-	-	7	13
19[c]	7.46	4.16	59.55	5.04	0.58	1.40	3.99	1.02	7.48	3.61	177.90	4.56	0.98	1.50	3.57	1.03	17	13
20	7.46	5.07	118.36	6.12	0.89	1.20	3.91	1.09	7.41	5.29	90.42	5.54	1.50	1.40	4.61	1.07	20	14
21	7.44	4.12	104.39	4.73	1.17	1.10	-	-	7.37	4.34	156.58	4.12	1.58	1.20	-	-	24	22
22	7.46	4.01	149.97	4.82	1.41	1.90	-	-	7.28	5.04	78.66	3.86	2.13	2.20	-	-	16	20
23	7.44	4.27	202.90	4.84	1.02	1.40	3.92	1.17	7.36	4.74	236.71	4.40	1.60	1.30	3.77	1.19	20	14
24	7.35	5.04	114.68	4.58	1.92	1.40	-	-	7.30	4.56	99.98	3.67	2.80	2.40	-	-	18	14
25	7.30	5.18	197.02	4.16	2.89	2.00	-	-	7.24	6.17	57.34	4.29	2.84	1.90	-	-	12	12
26	7.39	6.28	109.54	6.28	1.63	1.90	-	-	7.25	8.14	116.89	5.68	2.25	1.80	-	-	22	21
27	7.32	5.55	121.30	4.68	2.14	1.70	6.92	1.08	6.96	9.38	116.89	3.22	4.59	1.90	4.73	1.19	12	18
28	7.25	5.18	179.37	3.63	3.13	2.40	8.46	0.93	7.15	6.64	43.37	3.65	2.87	2.40	5.35	1.00	7	8
29	7.37	3.65	207.31	3.50	3.03	2.40	-	-	7.11	7.99	143.35	3.94	3.88	2.10	-	-	20	14
30	7.35	4.49	144.82	4.08	1.74	-	2.85	1.19	-	-	-	-	-	2.00	-	-	22	12
31	7.56	3.47	77.19	5.31	0.76	1.70	-	-	7.32	4.96	55.14	4.23	2.02	1.70	-	-	12	14
32	7.32	5.57	83.08	4.74	1.72	1.70	4.12	1.13	7.20	6.14	51.74	3.86	1.87	1.60	4.50	1.06	19	15
33	7.44	4.08	190.21	4.72	1.60	1.40	4.22	1.00	7.40	3.55	216.45	3.68	2.19	1.60	4.22	1.02	24	16
34	7.33	5.21	123.89	4.54	1.09	1.80	4.67	1.20	7.24	3.90	171.26	2.69	2.66	1.90	-	-	20	28

Supporting tables

ID	pH (1)	PCO_2 (1)	PO_2 (1)	HCO_3^- (1)	Lac (1)	Glu (1)	K (1)	Mg (1)	pH (2)	PCO_2 (2)	PO_2 (2)	HCO_3^- (2)	Lac (2)	Glu (2)	K (2)	Mg (2)	HR	RR
35	7.49	3.83	56.85	5.00	0.63	1.40	7.76	1.07	7.46	4.15	122.44	4.97	1.07	2.10	-	-	21	20
36	7.38	4.68	110.77	4.60	1.93	2.00	3.86	1.17	7.26	6.10	73.61	4.47	2.38	2.30	-	-	12	10
37	7.39	4.14	59.70	4.14	2.30	0.90	6.31	1.12	7.23	5.38	100.48	3.66	3.10	0.80	6.11	1.61	24	16
38	7.31	4.70	70.63	3.93	2.34	2.10	-	-	7.11	5.87	82.28	2.92	3.81	2.00	-	-	14	16
39	7.41	4.61	147.16	4.88	1.30	1.80	4.71	1.01	7.22	6.59	93.25	4.38	2.18	1.80	6.03	1.05	20	15
40	7.19	5.74	80.13	3.51	2.56	1.70	-	-	7.07	8.01	67.02	3.63	3.76	2.00	-	-	16	20
41	7.20	5.80	37.86	3.62	1.98	1.30	5.13	1.09	-	-	-	-	-	-	-	-	-	-
42	7.48	4.60	59.70	5.83	0.95	1.70	5.98	1.00	7.28	6.97	64.80	5.34	1.51	1.90	3.97	0.96	18	13
43	7.39	4.28	161.64	4.33	1.60	1.30	5.15	1.25	7.39	4.07	191.49	4.07	1.78	1.40	4.64	1.29	24	16
44	7.30	5.30	104.12	4.27	2.22	1.70	5.01	1.09	7.34	4.70	98.29	4.21	2.16	2.30	4.18	0.96	18	6
45	7.53	3.44	173.06	5.19	0.68	1.20	4.72	1.01	7.45	4.21	106.29	5.15	1.03	0.60	5.67	1.06	10	10
46	7.49	4.05	170.11	5.45	1.51	1.30	5.13	0.84	7.64	2.21	213.66	4.36	1.26	1.40	-	-	6	8
47	7.38	5.54	-	5.72	-	1.10	-	-	7.50	3.32	173.12	4.57	1.10	1.50	-	-	10	11
48	7.50	3.85	209.78	5.38	0.59	1.30	-	-	7.56	2.90	209.78	4.65	0.68	1.60	-	-	9	14
49	7.58	4.19	190.09	7.06	0.41	0.70	8.08	0.84	7.66	2.36	180.58	4.95	0.48	1.40	-	-	8	-
50	7.43	3.50	154.08	3.90	0.99	1.60	4.35	1.01	7.40	3.32	159.92	3.44	1.23	1.80	5.43	1.11	24	19
51	7.37	3.59	170.15	3.42	2.25	1.60	-	-	7.13	6.14	99.31	3.22	3.36	1.90	-	-	13	6
52[b]	7.36	4.67	132.85	4.36	2.01	1.90	-	-	7.52	-	152.56	-	1.99	1.60	-	-	-	12
53	7.38	4.81	214.61	4.77	1.68	1.30	4.32	1.04	7.36	4.31	121.90	4.03	2.03	1.10	8.51	1.10	-	12
54	7.44	3.68	124.87	4.26	1.32	1.50	-	-	7.38	4.30	174.53	4.19	1.74	1.70	-	-	-	16
55	7.45	5.11	48.93	5.96	1.41	1.40	-	-	7.42	4.28	110.27	4.67	1.60	1.80	-	-	16	14
56	7.32	5.67	122.63	4.84	1.77	1.77	4.57	0.83	-	-	-	-	-	-	-	-	3	1
57	7.37	3.42	162.78	3.30	1.98	1.60	5.57	1.00	7.27	3.49	166.43	2.62	2.94	1.60	-	-	5	4.5

Supporting tables

ID	pH (1)	PCO_2 (1)	PO_2 (1)	HCO_3^- (1)	Lac (1)	Glu (1)	K (1)	Mg (1)	pH (2)	PCO_2 (2)	PO_2 (2)	HCO_3^- (2)	Lac (2)	Glu (2)	K (2)	Mg (2)	HR	RR
58	-	-	-	-	-	-	-	-	7.18	3.39	182.49	2.04	3.31	1.60	-	-	16	18
59[a]	-	-	154.12	-	1.80	1.70	-	-	7.18	4.78	123.73	2.83	2.57	1.80	-	-	20	20
60	7.42	3.98	75.25	4.32	1.78	1.00	-	-	7.43	2.67	149.77	2.98	2.31	1.60	-	-	20	16
61	7.40	3.50	200.42	3.66	2.41	1.90	-	-	7.26	5.58	177.99	4.13	2.75	2.00	-	-	22	16
62	7.41	4.24	139.07	4.51	1.47	1.70	-	-	-	-	-	-	-	-	-	-	12	-

Supporting tables

Table S4. Summary of analyses. For each section of our analysis, the sub-sections, details, figures and tables are provided. Abbreviations: blood sample one (BS1); blood sample two (BS2); generalised additive model (GAM); generalised linear model (GLM).

Section	Sub-section	Details	Figures	Tables
1. Capture fights	A. Fight time	I. Fight time ~ explanatory variables (GLM)	S2	S6
2. Blood parameters	A. Physiological state	I. Visualisation & summary statistics	1	S7
		II. BS1 ~ explanatory variables (GLM)	2, S3–10	S8
		III. BS2 ~ explanatory variables (GLM)	3, S11–16	S9
	B. Physiological change	I. Visualisation & summary statistics	1	S10
		II. Percentile bootstrap analysis	-	S10
		III. Change in blood parameter values from BS1 to BS1 ~ explanatory variables (GLM)	S17–21	S11
	C. Synthesis	I. Effect ratios analysis	2, 3, S21	-
3. Heart and respiratory rates	A. Heart rates	I. Heart rate ~ explanatory variables (GAM)	4, S22	S12
	B. Respiration rates	I. Respiration rate ~ explanatory variables (GAM)	S23–4	S13

Supporting tables

Table S5. Summary of ‘skate with heath impairments. Individual health status was classified from physical examination and the presence/absence of injuries/infections (see Supporting Information §1). The skate included in this table were excluded from analysis of blood parameters as only ‘healthy’ individuals were included in the study. For each event (ID), a description of the clinical signs observed, the anatomical location of the lesion and the outcome or recapture information is shown. Sorted by ID.

ID	Description of lesion/injury	Location of lesion/injury	Fate
10	Suspected coelomitis; yellow flocculent material observed in celom when placing tag. Aborted tag placement and incision closed. Antibiotics administered		Recaptured:<br>21-06-01<br>21-06-29<br>22-02-13<br>22-03-18
21	Hooked in body wall through celom. Treated (body wall repair with sutures and antibiotics administered)	Penetration of celom, midline to right caudal body wall	Unknown. Not caught again
29	Deep penetrating full-thickness wound to leading edge of left wing, ventral aspect	Cranioventral aspect of left wing	Recaptured:<br>20-08-17<br>22-04-25
	Trauma to oral cavity. Loss of teeth and tissue damage with scab/plaque formation	Mandible, second and third tooth rows right of midline	
36	Abscess<br>Lanced, drained	Head. Ventro-lateral aspect of right mandible and intra-oral	Recaptured:<br>19-09-17
41	Abscess<br>(Visible in photographs of prior captures: 17-06-17, 18-03-25, 18-05-04, 18-08-07, 19-06-08)<br>Lanced, drained	Head. Lateral to right eye	Recaptured:<br>21-05-18<br>21-09-16<br>No evidence of abscess recurrence
56	Abscess, 2 cm diameter<br>Lanced, drained	Body, dorsal midline	Unknown. Not caught again
	Abscess, diameter ≈ 7 cm<br>Lanced, drained	Caudal body, left pelvic fin	
	Ventral erythema	Caudal body, junction of left pelvic fin and tail	

Table S6. Coefficient estimates from a Gaussian generalised linear model of fight time in relation to sex, body size (dorsal surface area), current speed, sun angle, bottom temperature and depth. For each coefficient, the estimate, standard error, z-value and p-value are shown. Estimates were derived from 35 observations. Deviance explained was 32 %.

**Coefficient**	**Estimate**	**SE**	***t*-value**	***p*-value**
Intercept	2.733	0.693	3.946	0.001
Sex_M	-0.105	0.180	-0.583	0.565
Size	0.644	0.528	1.219	0.233
Current speed	-0.764	3.869	-0.198	0.845
Sun angle	0.002	0.012	0.163	0.872
Temperature	-0.039	0.037	-1.060	0.299
Depth	0.001	0.002	0.707	0.485
Size: Current speed	0.516	3.421	0.151	0.881

Table S7. A summary of blood parameters. For each parameter, the name, number of observations and the minimum, median and maximum observed values for blood sample one and blood sample two are given. Note that the number of observations (N_{obs}) differs from the number of observations available for model fitting because not all observations were accompanied by the full suite of explanatory variables (Tables S3, S8–9 and S11).

Parameter	Units	N_{obs} [1]	N_{obs} [2]	Min [1]	Min [2]	Med [1]	Med [2]	Max [1]	Max [2]
pH	-	50	44	7.19	6.96	7.41	7.36	7.58	7.66
PCO_2	mmHg	50	43	3.42	2.21	4.25	4.56	6.57	9.38
PO_2	mmHg	50	44	45.23	43.37	128.86	123.08	216.77	236.71
HCO_3^-	$mmolL^{-1}$	50	43	3.30	2.04	4.73	4.19	7.06	5.76
Lac	$mmolL^{-1}$	50	44	0.41	0.48	1.31	1.93	3.13	4.59
Glu	$mmolL^{-1}$	50	44	0.70	0.60	1.60	1.80	2.50	2.60
K	$mmolL^{-1}$	28	18	2.85	3.57	4.72	4.62	8.46	8.51
Mg	$mmolL^{-1}$	28	18	0.79	0.96	1.02	1.06	1.25	1.61

Table S8. Coefficient estimates from blood parameter models at blood sample one. For each blood parameter, the number of observations available for model fitting, coefficient estimates, standard errors, *t*-values, *p*-values and the % deviance explained are shown. Note that the number of observations for each parameter may differ from the number of observations available for modelling fitting due to missing observations for explanatory variables (Tables S3 and S7).

Par.	N	Coefficient	Estimate	SE	*t*-value	*p*-value	D (%)
pH	48	Intercept	2.018	0.012	166.614	0.000	53.409
		Sex_M	-0.002	0.002	-1.013	0.317	
		Size	0.000	0.000	1.034	0.308	
		Temperature	-0.001	0.001	-0.582	0.564	
		Time (hook → surface)	0.000	0.000	0.366	0.717	
		Time (surface → BS1)	-0.001	0.000	-3.066	0.004	
		$Gaff_Y$	0.003	0.004	0.780	0.440	
		Temperature: Time (hook → surface)	0.000	0.000	-1.879	0.068	
PCO_2	48	Intercept	1.165	0.267	4.365	0.000	21.860
		Sex_M	0.006	0.049	0.127	0.900	
		Size	0.001	0.001	0.664	0.510	
		Temperature	0.010	0.021	0.474	0.638	
		Time (hook → surface)	0.001	0.008	0.185	0.854	
		Time (surface → BS1)	0.002	0.005	0.393	0.696	
		$Gaff_Y$	-0.127	0.074	-1.715	0.094	
		Temperature: Time (hook → surface)	0.000	0.001	0.520	0.606	
PO_2	48	Intercept	5.301	0.622	8.525	0.000	10.060
		Sex_M	-0.090	0.120	-0.749	0.458	
		Size	-0.002	0.003	-0.473	0.639	
		Temperature	-0.031	0.049	-0.634	0.530	
		Time (hook → surface)	0.000	0.018	0.015	0.988	
		Time (surface → BS1)	-0.003	0.012	-0.244	0.809	
		$Gaff_Y$	0.045	0.218	0.204	0.839	
		Temperature: Time (hook → surface)	0.001	0.002	0.517	0.608	
HCO_3^-	48	Intercept	1.641	0.208	7.870	0.000	37.673
		Sex_M	-0.035	0.042	-0.830	0.411	
		Size	0.002	0.001	1.825	0.075	
		Temperature	-0.010	0.017	-0.574	0.569	
		Time (hook → surface)	0.004	0.007	0.589	0.559	
		Time (surface → BS1)	-0.012	0.005	-2.573	0.014	
		$Gaff_Y$	-0.066	0.061	-1.069	0.292	

Supporting tables

Par.	N	Coefficient	Estimate	SE	*t*-value	*p*-value	D (%)
		Temperature: Time (hook → surface)	-0.001	0.001	-1.278	0.209	
Lac	48	Intercept	-0.909	0.701	-1.297	0.202	66.333
		Sex_M	0.166	0.085	1.946	0.059	
		Size	-0.002	0.002	-0.855	0.398	
		Temperature	0.080	0.052	1.532	0.133	
		Time (hook → surface)	-0.006	0.021	-0.271	0.788	
		Time (surface → BS1)	0.014	0.009	1.622	0.113	
		$Gaff_Y$	0.151	0.150	1.008	0.319	
		Temperature: Time (hook → surface)	0.002	0.002	1.094	0.281	
Glu	48	Intercept	0.443	0.320	1.387	0.173	38.544
		Sex_M	0.064	0.056	1.143	0.260	
		Size	-0.002	0.001	-1.450	0.155	
		Temperature	0.004	0.024	0.169	0.866	
		Time (hook → surface)	-0.001	0.009	-0.140	0.889	
		Time (surface → BS1)	0.001	0.006	0.158	0.875	
		$Gaff_Y$	0.250	0.092	2.718	0.010	
		Temperature: Time (hook → surface)	0.001	0.001	0.926	0.360	
K	27	Intercept	3.219	0.513	6.276	0.000	38.606
		Sex_M	0.071	0.100	0.705	0.489	
		Size	-0.002	0.003	-0.709	0.487	
		Temperature	-0.104	0.037	-2.784	0.012	
		Time (hook → surface)	-0.041	0.019	-2.090	0.050	
		Time (surface → BS1)	-0.030	0.022	-1.388	0.181	
		$Gaff_Y$	-0.066	0.201	-0.330	0.745	
		Temperature: Time (hook → surface)	0.004	0.001	2.697	0.014	
Mg	27	Intercept	-0.122	0.183	-0.666	0.513	60.052
		Sex_M	-0.016	0.033	-0.473	0.642	
		Size	-0.001	0.001	-1.771	0.093	
		Temperature	0.029	0.014	2.107	0.049	
		Time (hook → surface)	0.007	0.006	1.184	0.251	
		Time (surface → BS1)	0.008	0.006	1.340	0.196	
		$Gaff_Y$	-0.065	0.069	-0.945	0.357	
		Temperature: Time (hook → surface)	-0.001	0.000	-1.011	0.325	

Table S9. Coefficient estimates from blood parameter models at blood sample two. As in Table S6, for each blood parameter, the number of observations available for model fitting, coefficient estimates, standard errors, *t*-values, *p*-values and the % deviance explained are shown. Note that the number of observations for each parameter may differ from the number of observations available for modelling fitting due to missing observations for explanatory variables (Tables S3 and S7). Unlike BS1, potassium and magnesium were not modelled at BS2 given the limited number of observations.

Par.	N	Coefficient	Estimate	SE	*t*-value	*p*-value	D (%)
pH	42	Intercept	2.042	0.031	65.792	0.000	47.049
		Sex_M	0.000	0.006	-0.019	0.985	
		Size	0.000	0.000	1.566	0.127	
		Temperature	-0.006	0.003	-2.232	0.033	
		Time (hook → surface)	-0.001	0.001	-0.665	0.511	
		Time (surface → BS2)	0.000	0.001	0.384	0.703	
		$Gaff_Y$	-0.013	0.008	-1.610	0.117	
		$Surgery_Y$	-0.004	0.010	-0.456	0.651	
		Temperature: Time (hook → surface)	0.000	0.000	0.107	0.916	
PCO_2	41	Intercept	0.489	0.762	0.642	0.526	33.860
		Sex_M	0.012	0.099	0.120	0.906	
		Size	-0.003	0.003	-1.045	0.304	
		Temperature	0.117	0.058	2.010	0.053	
		Time (hook → surface)	0.025	0.022	1.139	0.263	
		Time (surface → BS2)	-0.018	0.014	-1.252	0.220	
		$Gaff_Y$	0.232	0.156	1.487	0.147	
		$Surgery_Y$	0.155	0.167	0.926	0.361	
		Temperature: Time (hook → surface)	-0.001	0.002	-0.832	0.412	
PO_2	42	Intercept	5.448	0.635	8.579	0.000	30.827
		Sex_M Sex_M	-0.132	0.121	-1.085	0.286	
		Size	-0.001	0.003	-0.265	0.793	
		Temperature	-0.022	0.055	-0.403	0.690	
		Time (hook → surface)	0.009	0.022	0.412	0.683	
		Time (surface → BS2)	0.017	0.017	1.009	0.320	
		$Gaff_Y$	-0.228	0.169	-1.350	0.186	
		$Surgery_Y$	-0.116	0.199	-0.582	0.565	
		Temperature: Time (hook → surface)	-0.001	0.002	-0.706	0.485	
HCO_3^-	41	Intercept	1.595	0.352	4.527	0.000	34.373
		Sex_M	-0.065	0.065	-1.002	0.324	
		Size	0.002	0.001	1.094	0.282	

Supporting tables

**Par.**	**N**	**Coefficient**	**Estimate**	**SE**	***t*-value**	***p*-value**	**D (%)**
		Temperature	-0.008	0.028	-0.292	0.772	
		Time (hook → surface)	0.009	0.011	0.768	0.448	
		Time (surface → BS2)	-0.017	0.009	-1.835	0.076	
		$Gaff_Y$	-0.008	0.090	-0.092	0.927	
		$Surgery_Y$	0.065	0.109	0.595	0.556	
		Temperature: Time (hook → surface)	-0.001	0.001	-1.098	0.280	
Lac	42	Intercept	-1.315	1.250	-1.052	0.300	50.424
		Sex_M	0.116	0.118	0.983	0.333	
		Size	-0.003	0.004	-0.851	0.401	
		Temperature	0.168	0.097	1.736	0.092	
		Time (hook → surface)	0.014	0.038	0.368	0.715	
		Time (surface → BS2)	0.008	0.016	0.533	0.597	
		$Gaff_Y$	0.260	0.196	1.322	0.195	
		$Surgery_Y$	-0.036	0.219	-0.164	0.871	
		Temperature: Time (hook → surface)	-0.001	0.003	-0.201	0.842	
Glu	41	Intercept	-0.567	0.516	-1.098	0.280	26.270
		Sex_M	0.101	0.075	1.344	0.188	
		Size	0.000	0.002	-0.047	0.963	
		Temperature	0.066	0.041	1.612	0.117	
		Time (hook → surface)	0.023	0.016	1.453	0.156	
		Time (surface → BS2)	0.003	0.010	0.261	0.796	
		$Gaff_Y$	0.066	0.109	0.608	0.547	
		$Surgery_Y$	0.075	0.135	0.553	0.584	
		Temperature: Time (hook → surface)	-0.001	0.001	-1.167	0.252	

Table S10. Percentile bootstrap paired-samples test statistics for the change in blood parameter values from blood sample one (BS1) and two (BS2). For each comparison type (all individuals, untagged individuals and tagged individuals) and each parameter, the number of observations available for the bootstrap procedure (i.e., the number of individuals in that group with observations at both BS1 and BS1) is shown, alongside the mean observed difference, the mean simulated difference, the standard error (the standard deviation of the simulated differences), the bias (mean simulated difference – mean observed difference), the percent bias, the null hypothesis (a difference of zero), the alterative hypothesis (whether or not mean difference is less-than, greater-than or simply not-equal to zero), the *p*-value and the *p*-value adjusted by the Bonferroni Correction for multiple comparisons (separately for each group) is shown. Low bias scores demonstrate that the use of the percentile bootstrap method was appropriate. Units for PCO_2 and PO_2 are mmHg; units for HCO_3^-, lactate, glucose, K and Mg are $mmolL^{-1}$.

Type	Par.	N	MD_{obs}	MD_{sim}	SE	Bias	Bias (%)	H_0	H_1	*p*-value	*p*-value (BC)
BS1 vs. BS2	pH	42	-0.061	-0.061	0.017	0.000	0.231	0	less-than	0.000	0.001
	PCO_2	41	0.316	0.312	0.208	-0.004	1.390	0	greater-than	0.067	0.534
	PO_2	42	-0.815	-0.881	9.814	-0.066	8.030	0	not-equal	0.920	1.000
	HCO_3^-	41	-0.611	-0.611	0.086	0.000	0.012	0	less-than	0.000	0.000
	Lac	42	0.511	0.511	0.081	0.000	0.060	0	greater-than	0.000	0.000
	Glu	41	0.144	0.144	0.045	0.000	0.018	0	greater-than	0.000	0.003
	K	18	-0.193	-0.194	0.384	0.000	0.207	0	greater-than	0.698	1.000
	Mg	18	0.049	0.049	0.029	0.000	0.319	0	not-equal	0.049	0.394
BS1 vs. BS2 (untagged)	pH	7	-0.077	-0.077	0.020	0.000	0.056	0	less-than	0.000	0.000
	PCO_2	7	0.280	0.286	0.397	0.006	2.220	0	greater-than	0.243	1.000
	PO_2	7	5.275	5.409	20.135	0.135	2.560	0	not-equal	0.669	1.000
	HCO_3^-	7	-0.645	-0.645	0.225	0.000	0.001	0	less-than	0.000	0.000
	Lac	7	0.594	0.593	0.172	-0.001	0.214	0	greater-than	0.000	0.000
	Glu	7	0.057	0.058	0.045	0.000	0.847	0	greater-than	0.098	0.787

Supporting tables

Type	Par.	N	MD_{obs}	MD_{sim}	SE	Bias	Bias (%)	H_0	H_1	*p*-value	*p*-value (BC)
	K	4	0.093	0.096	0.297	0.004	4.020	0	greater-than	0.372	1.000
	Mg	4	0.163	0.163	0.096	0.000	0.222	0	not-equal	0.000	0.001
BS1 vs. BS2 (tagged)	pH	35	-0.058	-0.058	0.019	0.000	0.718	0	less-than	0.001	0.010
	PCO_2	34	0.323	0.323	0.238	-0.001	0.177	0	greater-than	0.088	0.702
	PO_2	35	-2.033	-1.938	10.974	0.095	4.690	0	not-equal	0.854	1.000
	HCO_3^-	34	-0.604	-0.602	0.094	0.002	0.260	0	less-than	0.000	0.000
	Lac	35	0.495	0.495	0.091	0.000	0.044	0	greater-than	0.000	0.000
	Glu	34	0.162	0.163	0.054	0.001	0.611	0	greater-than	0.001	0.007
	K	14	-0.275	-0.279	0.482	-0.004	1.560	0	greater-than	0.721	1.000
	Mg	14	0.016	0.017	0.018	0.000	0.968	0	not-equal	0.356	1.000

Table S11. Coefficient estimates from blood parameter models for the change between blood sample one (BS1) and two (BS2). As in Table S9, for each blood parameter, the number of observations available for model fitting, coefficient estimates, standard errors, *t*-values, *p*-values and the % deviance explained are shown. Note that the number of observations for each parameter may differ from the number of observations available for modelling fitting due to missing observations for explanatory variables (Tables S3 and S7). Unlike BS1, potassium and magnesium were not modelled at BS2 given the limited number of observations.

Par.	N	Coefficient	Estimate	SE	*t*-value	*p*-value	D (%)
pH	40	Intercept	0.207	0.182	1.135	0.265	39.101
		Sex$_{M}$	0.041	0.034	1.192	0.243	
		Size	0.001	0.001	1.616	0.116	
		Temperature	-0.042	0.015	-2.822	0.008	
		Time (hook → surface)	-0.008	0.006	-1.430	0.163	
		Time (surface → BS1)	0.004	0.005	0.752	0.458	
		Time (BS1 → BS2)	0.007	0.007	1.063	0.296	
		Gaff$_{Y}$	-0.117	0.048	-2.413	0.022	
		Surgery$_{Y}$	-0.076	0.063	-1.204	0.238	
		Temperature: Time (hook → surface)	0.001	0.000	1.510	0.141	
HCO_3^-	39	Intercept	-0.525	1.052	-0.500	0.621	17.941
		Sex$_{M}$	0.214	0.203	1.054	0.300	
		Size	-0.003	0.005	-0.584	0.564	
		Temperature	-0.008	0.086	-0.098	0.923	
		Time (hook → surface)	0.001	0.034	0.042	0.967	
		Time (surface → BS1)	0.022	0.029	0.767	0.449	
		Time (BS1 → BS2)	-0.019	0.040	-0.483	0.633	
		Gaff$_{Y}$	0.309	0.279	1.105	0.278	
		Surgery$_{Y}$	0.007	0.365	0.019	0.985	
		Temperature: Time (hook → surface)	0.001	0.003	0.274	0.786	
Lac	40	Intercept	-1.207	0.959	-1.259	0.218	33.027
		Sex$_{M}$	-0.097	0.181	-0.534	0.597	
		Size	-0.005	0.005	-0.942	0.354	
		Temperature	0.215	0.079	2.712	0.011	
		Time (hook → surface)	0.041	0.031	1.334	0.192	
		Time (surface → BS1)	-0.007	0.026	-0.254	0.801	
		Time (BS1 → BS2)	-0.003	0.035	-0.085	0.933	
		Gaff$_{Y}$	0.346	0.296	1.170	0.251	
		Surgery$_{Y}$	0.254	0.326	0.777	0.443	
		Temperature: Time (hook → surface)	-0.005	0.003	-1.777	0.086	

Supporting tables

**Par.**	**N**	**Coefficient**	**Estimate**	**SE**	***t*-value**	***p*-value**	**D (%)**
Glu	39	Intercept	-0.847	0.555	-1.527	0.138	36.004
		Sex_M	0.096	0.099	0.965	0.343	
		Size	0.003	0.002	1.173	0.250	
		Temperature	0.034	0.046	0.744	0.463	
		Time (hook → surface)	0.010	0.019	0.522	0.606	
		Time (surface → BS1)	0.019	0.015	1.282	0.210	
		Time (BS1 → BS2)	0.012	0.019	0.648	0.522	
		$Gaff_Y$	-0.360	0.143	-2.521	0.017	
		$Surgery_Y$	0.101	0.177	0.572	0.572	
		Temperature: Time (hook → surface)	-0.001	0.002	-0.880	0.386	

Table S12. Coefficient estimates from a negative binomial generalised additive model of heart rate in relation to sex, body size (total length), bottom temperature, fight time, time at the water surface, gaffing, surgery and time on deck. For parametric coefficients, the estimate, standard error and *z*-value are shown. For smooth terms, the effective degrees of freedom, reference degrees of freedom and χ^2 statistic are shown. Estimates were derived from 149 observations. Deviance explained was 82 %.

**Parametric coefficients**				
Term	***Estimate***	***SE***	***z-value***	***p-value***
Intercept	2.332	0.436	5.348	0.000
Sex_M	0.069	0.072	0.967	0.334
Size	-0.006	0.002	-3.277	0.001
Temperature	0.120	0.034	3.516	0.000
Time (hook → surface)	0.021	0.013	1.585	0.113
Time (surface → deck)	0.020	0.016	1.224	0.221
$Gaff_Y$	-0.016	0.101	-0.160	0.873
$Surgery_Y$	-0.081	0.113	-0.710	0.477
Temperature: Time (hook → surface)	-0.002	0.001	-1.851	0.064
**Approximate significance of smooth terms**				
Term	***EDF***	***Ref DF***	χ^2	***p-value***
s(event)	16.780	36	37.618	0.000
s(event, time [deck → observation])	1.649	45	2.202	0.299

Table S13. Coefficient estimates from a negative binomial generalised additive model of respiration rate in relation to sex, body size (total length), bottom temperature, fight time, time at the water surface, gaffing, surgery and time on deck. For parametric coefficients, the estimate, standard error and *z*-value are shown. For smooth terms, the effective degrees of freedom, reference degrees of freedom and χ^2 statistic are shown. Estimates were derived from 196 observations. Deviance explained was 75 %.

**Parametric coefficients**				
Term	***Estimate***	***SE***	***z-value***	***p-value***
Intercept	2.895	0.593	4.885	0.000
Sex_M	-0.018	0.097	-0.188	0.851
Size	-0.005	0.003	-1.893	0.058
Temperature	0.056	0.047	1.188	0.235
Time (hook → surface)	0.007	0.018	0.384	0.701
Time (surface → deck)	-0.053	0.025	-2.085	0.037
$Gaff_Y$	0.028	0.142	0.201	0.841
$Surgery_Y$	0.003	0.160	0.018	0.986
Temperature: Time (hook → surface)	-0.001	0.002	-0.466	0.641
**Approximate significance of smooth terms**				
Term	***EDF***	***Ref DF***	χ^2	***p-value***
s(event)	29.130	37	146.989	0.000
s(event, time [deck → observation])	0.002	46	0.002	0.556
